# Bright red aggregation-induced emission nanoparticles for multifunctional applications in cancer therapy†

**DOI:** 10.1039/c9sc06310b

**Published:** 2020-01-29

**Authors:** Liping Zhang, Weilong Che, Zhiyu Yang, Xingman Liu, Shi Liu, Zhigang Xie, Dongxia Zhu, Zhongmin Su, Ben Zhong Tang, Martin R. Bryce

**Affiliations:** Key Laboratory of Nanobiosensing and Nanobioanalysis at Universities of Jilin Province, Department of Chemistry, Northeast Normal University 5268 Renmin Street Changchun Jilin Province 130024 P. R. China zhudx047@nenu.edu.cn zmsu@nenu.edu.cn; State Key Laboratory of Polymer Physics and Chemistry, Changchun Institute of Applied Chemistry Chinese Academy of Sciences Changchun 130022 P. R. China xiez@ciac.ac.cn; State Key Laboratory of Molecular Neuroscience Institute for Advanced Study Institute of Molecular Functional Materials, The Hong Kong University of Science and Technology Clear Water Bay Kowloon Hong Kong China tangbenz@ust.hk; Department of Chemistry, Durham University Durham DH1 3LE UK m.r.bryce@durham.ac.uk

## Abstract

Developing multifunctional photosensitizers (PSs) is needed to effectively simplify cancer treatment, but it remains a big challenge. Here, two red-emitting AIE-active, donor–acceptor (D–A) PSs with small Δ*E*_ST_ and their AIE nanoparticles, are rationally designed and synthesized. The **PS1** NPs exhibit bright red-emission with high quantum yield, appropriate ^1^O_2_ generation ability and good biocompatibility. More importantly, **PS1** NPs can strongly light up the cytoplasm by gently shaking the cells for only 5 s at room temperature, indicating ultrafast staining and mild incubation conditions. *In vitro* and *in vivo* cell tracing demonstrate that **PS1** NPs can track cells over 14 days, and effectively inhibit tumor growth upon irradiation. To the best of our knowledge, this work is the first example of a PS that integrates image-guided PDT, ultrafast staining and long-term tracing functions, demonstrating the “all-in-one” concept which offers great advantages for potential clinical applications.

## Abstract 2

Developing multifunctional photosensitizers (PSs) is needed to effectively simplify cancer treatment, but it remains a big challenge.

## Graphical abstract

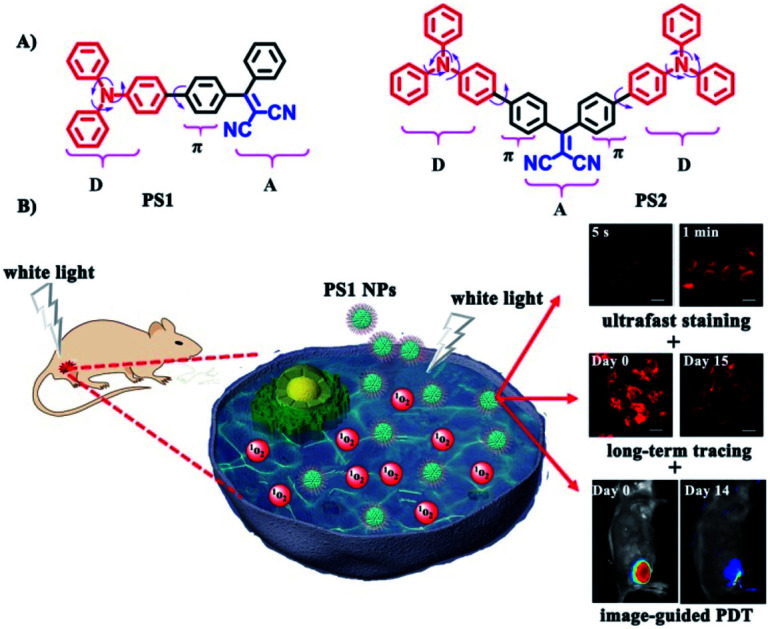

## Introduction

Image-guided photodynamic therapy (PDT) has recently gained increasing attention. This technique simultaneously achieves real-time molecular diagnosis and concurrent light-triggered therapy, and has surpassed traditional surgery, such as radiotherapy and chemotherapy.^1–8^ Noninvasive fluorescence imaging has emerged as a very powerful tool for visualizing clinical diagnostics and for biological research.^9–14^ Unfortunately, discontinuous and short-term cellular tracing is unable to provide continuous real-time dynamic information, thereby limiting the insights into a variety of complex biological processes.^15–21^ Long incubation times and harsh incubation conditions are critical barriers for cellular fluorescence imaging in clinical applications.^22–25^ Cancer treatments mainly include three essential steps: ultrafast staining, therapy, and long-term tracing. The former two play important roles for the discovery and excision of tumors, and the last is to further monitor the metastasis of residual tumor.^15,21^ The current scope of photosensitizers (PSs) is far from ideal, and until now, only a few PSs can achieve partial procedures, whereas there are no reports on simultaneous multiple applications, namely: (i) image-guided PDT, (ii) ultrafast staining and (iii) long-term tracking. Is it possible to design a single “all-in-one” PS that embraces all the above functions at the same time?

PSs with efficient generation of singlet oxygen (^1^O_2_) and bright red-emission are crucial to realize imaging-guided PDT.^26^ An effective strategy to improve the efficiency of ^1^O_2_ generation is to accelerate the intersystem crossing (ISC) and to reduce Δ*E*_ST_ (the energy gap between S_1_ and T_1_ states).^27–31^ A small Δ*E*_ST_ is obtained by constructing a conjugated donor–acceptor (D–A) structure, which is beneficial for strong intramolecular charge transfer (ICT) with efficient separation of the highest occupied molecular orbital (HOMO) and lowest unoccupied molecular orbital (LUMO), leading to red-shifted emission.^32–35^ Red emission is important because of its minimized autofluorescence interference, increased penetration depth, and less damage to tissue.^36–38^ However, traditional organic molecules tend to form aggregates in aqueous media, for example by π–π stacking, which directly leads to reduced ^1^O_2_ production, non-radiative pathways and quenched fluorescence.^27,39–41^

To overcome this challenge, the concept of aggregation-induced emission (AIE) has been exploited, notably by Tang's group.^42–45^ A series of AIEgen-based PSs have been reported to improve both the fluorescence intensity and the ^1^O_2_ production ability in the aggregated state, due to the restriction of intramolecular motions (RIMs) which lead to energy dissipation.^46–54^ Recently, our group obtained three red-emitting AIEgen nanoparticles (NPs) with higher brightness, enhanced ^1^O_2_ generation, and better biocompatibility compared to the pure iridium(iii) complexes.^55^ These precedents make AIE NPs with purely organic D–A units particularly suitable as smart PSs to successfully obtain an “all-in-one” (image-guided PDT, ultrafast staining and long-term tracing) system.

**Fig. 1:**
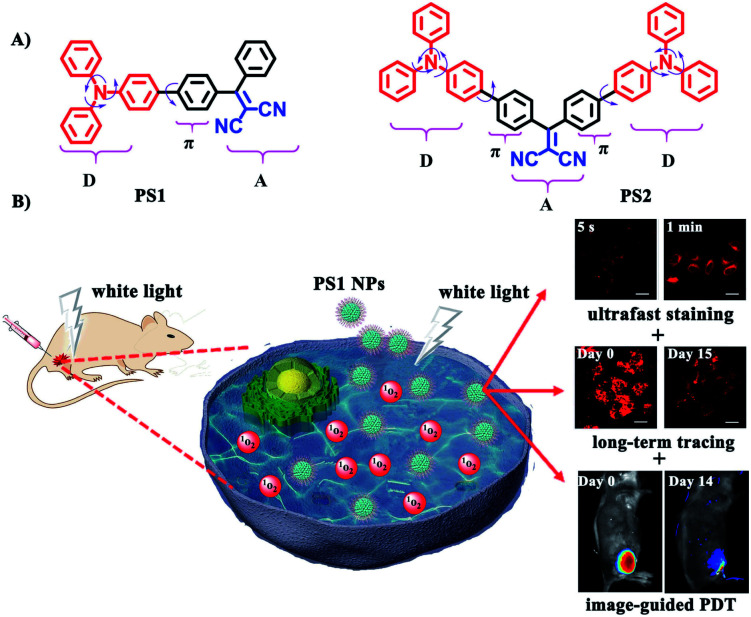
(A) Structural formulas of **PS1** and **PS2**. (B) Schematic illustration of **PS1** NPs as PSs for “all-in-one” PDT.

In this contribution new PSs are designed and easily synthesized in high yield *via* a three-bladed propeller-like triphenylamine (TPA) as the strong donor and the dicyanovinyl (DC) groups as the acceptor, in which TPA also acts as a rotor to realize AIE.^56^ The two red-emitting AIE D–A and D–A–D molecules are **PS1** and **PS2** (Fig. 1A) and their corresponding polymer-encapsulated NPs are **PS1** NPs and **PS2** NPs. **PS1** NPs possess bright red-emission, a large Stokes shift, appropriate ^1^O_2_ generation ability, good biocompatibility and excellent image-guided PDT activity. More importantly, **PS1** NPs achieve ultrafast staining of cells in only 5 s at room temperature, with excellent long-term imaging of more than 14 days *in vitro* and *in vivo*.

## Results and discussion

**Fig. 2:**
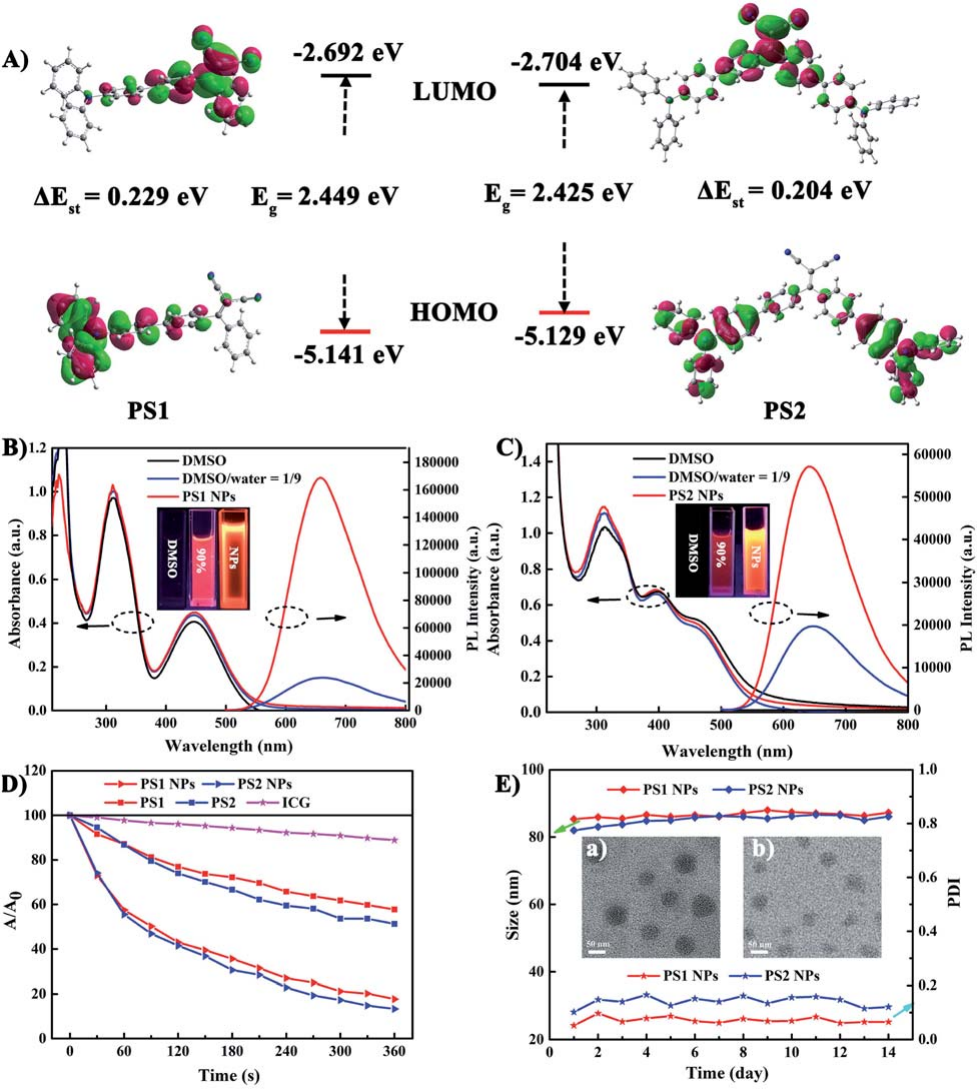
(A) HOMO and LUMO distributions of **PS1** and **PS2** based on TD-DFT. UV-vis and emission spectroscopies of (B) **PS1** and (C) **PS2** in DMSO, DMSO/water (v/v) = 1/9, and the corresponding PS NPs in water (*λ*_ex_ = 488 nm), insert: the fluorescent image of PSs and their NPs under 365 nm UV light. (D) The decomposition rates of ICG with different PSs under white irradiation (20 mW cm^−2^). (E) Size measurement of different PSs in 14 days, insert: the TEM images of (a) **PS1** NPs and (b) **PS2** NPs.

To gain an insight into the molecular properties of **PS1** and **PS2**, time-dependent density theory (TD-DFT) calculations were performed (Fig. 2A). The HOMOs are mainly localized on TPA units, whereas the LUMOs are located on DC units, with significant HOMO–LUMO separation. The energy gap between the HOMO and LUMO of **PS1** and **PS2** is 2.449 and 2.425 eV, respectively. These relatively narrow band gaps induce the red-shifted absorption. Furthermore, the additional triphenylamine unit in **PS2** results in a slight reduction of Δ*E*_ST_ (**PS2** 0.204 eV; **PS1** 0.229 eV), owing to a smaller spatial overlap of the Frontier orbitals. The small Δ*E*_ST_ values of **PS1** and **PS2** are beneficial for ISC, which will facilitate highly efficient ^1^O_2_ generation.^29^

The photophysical properties of the PSs were investigated by absorption and photoluminescence (PL) spectroscopy. In Fig. 2B and C the absorption bands at about 210–315 nm are assigned to the π–π* transition of the conjugated backbone. The bands located in the 380–600 nm region belong to the intense ICT process between the D–A units. The absorption band covers most of the visible light range, which is beneficial for matching the white light spectrum.^28^ In Fig. S9 (ESI†), both **PS1** and **PS2** show negligible fluorescence in DMSO, because the rotational motions of the triphenylamine moieties increase the dissipation of energy. However, the PL intensities of **PS1** and **PS2** gradually increase upon raising the water content, when intramolecular rotation is restricted by the formation of aggregates, revealing a typical AIE effect. Compared with **PS1** (*λ*_max_ 660 nm) and **PS2** (645 nm), their NPs show similar emission peaks but with significantly enhanced intensities. The PL intensities of **PS1** NPs and **PS2** NPs are 6.9 and 2.86 times higher than those of **PS1** and **PS2**, respectively, with **PS1** NPs 2.9 times higher than **PS2** NPs in the same conditions. The fluorescence spectra of these AIE NPs extend into the near-infrared region, and they exhibit a large Stokes shift which can eliminate interference from the background. These factors are favorable for bioimaging. The fluorescence quantum yields (QYs) of **PS1** NPs and **PS2** NPs in water are 40% and 32%, respectively, which are much higher than **PS1** and **PS2** (16% and 13%, respectively) in DMSO-water mixtures. Compared with tetraphenylethylene (TPE),^41^ in the present work TPA with stronger electron donating ability promoted a red-shift in the absorption and emission of the PSs. This shift is beneficial for efficient biological applications, such as image-guided PDT, ultrafast staining and long-term tracking. These results confirm that the AIE NPs possess favourable photophysical properties.

Photosensitized ^1^O_2_ generation plays an important role in PDT.^57^ To evaluate the ^1^O_2_ generation ability of **PS1**, **PS2** and their NPs, indocyanine green (ICG) was used as an indicator. No obvious changes of absorbance were observed in three control groups: (i) ICG + irradiation, (ii) PSs + irradiation, and (iii) ICG + PSs without irradiation (ESI, Fig. S14–S16†), verifying that all the PSs show good photostability.^58^ As expected, upon irradiation of ICG (5 μg mL^−1^) solutions in the presence of **PS1**, **PS2** and their NPs (30 μg mL^−1^), the absorption peak of ICG at 790 nm gradually decreases in intensity (Fig. 2D; ESI, Fig. S17†), indicating that the PSs possess ^1^O_2_ generation ability. In Fig. S18 (ESI†) all the PSs follow a first-order ^1^O_2_ generation behavior. The slopes can be ordered as **PS1** (1.45 × 10^−3^) < **PS2** (1.88 × 10^−3^) < **PS1** NPs (4.36 × 10^−3^) < **PS2** NPs (5.32 × 10^−3^). The slopes of the kinetic decays of **PS1** NPs and **PS2** NPs are 3.01 and 2.83 times higher than for **PS1** and **PS2**, respectively. A steeper slope represents a quicker decay rate of ICG and increased ability to generate ^1^O_2_. The ^1^O_2_ quantum yields of **PS1**, **PS2**, **PS1** NPs and **PS2** NPs are 38%, 43%, 67% and 76% with methylene blue (MB) as the reference (*Φ*Δ = 52% in MeCN). Overall, these NPs generate ^1^O_2_ very effectively and they possess great potential as PSs for PDT applications.

In addition, the morphology, size and stability of these NPs are important premises for biomedical applications. **PS1** NPs and **PS2** NPs are spherical and possess uniform sizes of 52 nm and 46 nm, respectively (Fig. 2E, insert). The corresponding hydrodynamic sizes are 82 nm and 79 nm, respectively (ESI, Fig. S19†). In addition, the diameters of **PS1** NPs and **PS2** NPs are stable within 14 days in water (Fig. 2E). Meanwhile, the fluorescence and absorbance intensity of **PS1** NPs and **PS2** NPs both retain more than 80% and 98%, respectively, of their initial values after 7 days (ESI, Fig. S11 and S12†), indicating that these NPs have excellent optical stability. The combination of spherical morphology, suitable size, superior physical stability and excellent photostability suggest that these NPs are suitable for the ensuing biological applications.

**Fig. 3:**
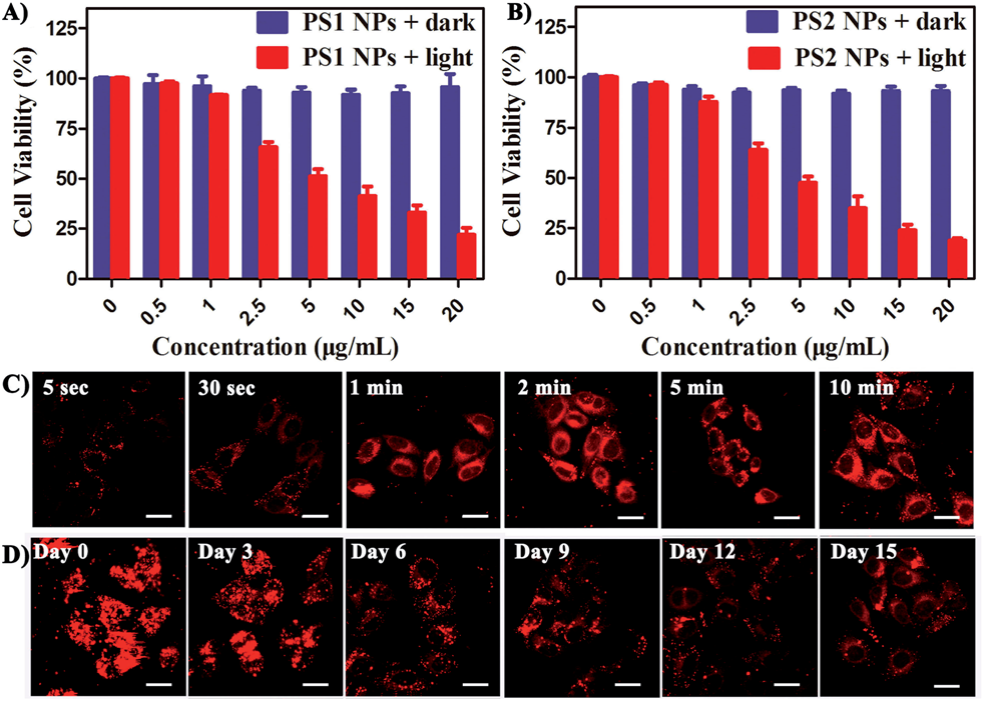
Viability of HeLa cells treated with (A) **PS1** NPs and (B) **PS2** NPs with or without white light (20 mW cm^−2^, 60 min). (C) CLSM images of HeLa cells after incubation with **PS1** NPs (3 μg mL^−1^) for different times. (D) Long-term cell tracing images of the **PS1** NPs (20 μg mL^−1^) at 37 °C for 6 h and then subcultured for a different number of days. Scale bar = 20 μm for all images.

*In vitro* cytotoxicity of all the PSs toward HeLa cells was measured by MTT colorimetric assay.^59^ The cells retained >95% viability after incubation with **PS1** NPs and **PS2** NPs for 24 h (Fig. 3A and B) indicating good cytocompatibility. Under white-light irradiation **PS1** NPs and **PS2** NPs have dose-dependent cytotoxicity. The half-maximal inhibitory concentration (IC_50_) is in the sequence **PS1** NPs (5.72 μg mL^−1^) > **PS2** NPs (4.31 μg mL^−1^). In contrast, **PS1** and **PS2** exhibit some dark cytotoxicity and low phototoxicity (ESI, Fig. S20†). The same results were observed in A549 cells and MDA-MB-231 cells (ESI, Fig. S21†). Intracellular ^1^O_2_ generation of **PS2** NPs was further investigated by using DCFH-DA as indicator. In Fig. S23 (ESI†) after irradiation the green emission from **PS2** NPs is evidently brighter than that from **PS2**, indicating the enhanced ^1^O_2_ generation ability of the nanoparticle formulation. Moreover, the anticancer efficiencies of **PS1**, **PS2** and their NPs were examined by live/dead staining techniques (ESI, Fig. S22†). Under light irradiation, negligible green fluorescence has been observed which indicates that the cancer cells are completely killed. The proportion of dead cells in the group of **PS1** NPs and **PS2** NPs under irradiation was much higher than with **PS1** and **PS2**, which agrees with the above MTT results.

The bright fluorescence emission, large Stokes shift, favorable stability, and good cytocompatibility of **PS1** NPs and **PS2** NPs inspired us to conduct further investigation in their intracellular imaging behavior. HeLa cells were incubated with **PS1** NPs and **PS2** NPs (3 μg mL^−1^) for different times as depicted in Fig. 3C and S28 (ESI†). Bright red emission was observed from the cells treated with **PS1** NPs, whereas negligible emission was observed with **PS2** NPs. The **PS1** NPs are more suitable for ultrafast staining, because as described above that **PS1** NPs possess brighter red emission and higher fluorescence quantum yield (40%) than **PS2** NPs. The cytoplasm was brightly emissive after simply shaking the cell incubate with **PS1** NPs for 5 s at room temperature, demonstrating its ultrafast staining on the timescale of a few seconds. More strikingly, the cellular interior was lit up by incubation with 0.5 μg mL^−1^ of **PS1** NPs (ESI, Fig. S24†). These results strongly validate that the **PS1** NPs could be applied for ultrafast staining.

The applications of the **PS1** NPs and **PS2** NPs for *in vitro* long-term cellular tracing were investigated. As shown in Fig. 3D and S31 (ESI†), the internal cells were stained by **PS1** NPs and **PS2** NPs with bright red fluorescence in their initial stages which gradually decreased with time. Obviously, the cell tracking ability of **PS1** NPs is better than that of **PS2** NPs, which could be attributed to the brighter red emission of **PS1** NPs than **PS2** NPs. Negligible change in cell morphology was observed from day 1 to day 15 during long-term cell tracing. This indicates that there are no cytotoxicity effects for long-term cell tracing with **PS1** and **PS2** NPs. Under the same conditions, flow cytometry results also demonstrate that both **PS1**NPs and **PS2** NPs have a tendency to accumulate in HeLa cells after 15 days incubation (ESI, Fig. S33†). Therefore, the **PS1** NPs could perform as a promising long-term tracing probe.

**Fig. 4:**
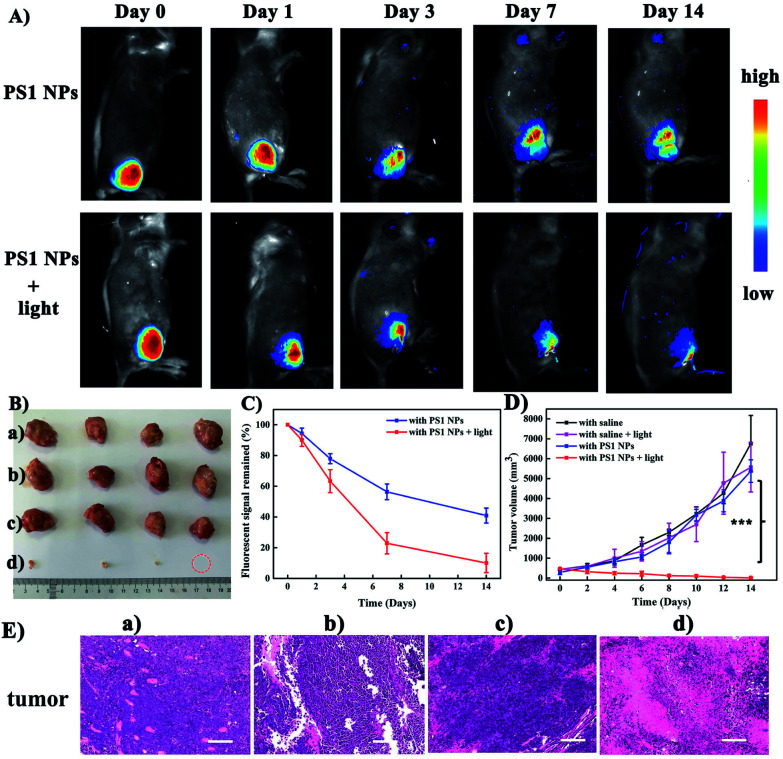
(A) Time-dependent *in vivo* fluorescence images of U14 tumor-bearing mice after intratumoral injection with **PS1** NPs (100 μg mL^−1^, 100 μL) or with **PS1** NPs + white light (200 mW cm^−2^, 20 min). (B) Harvested tumors from various groups treated. (C) Fluorescence intensity of U14 tumor-bearing mice in different mice groups. (D) Tumor volume measurement for different treatments of mice (***P < 0.001, *n* = 4 per group, PDT *vs.* other groups). (E) H&E staining images of tumor slices from each groups. Scale bars: 100 μm. (a) With saline, (b) with saline and light, (c) with **PS1** NPs, (d) with **PS1** NPs and light (100 μg mL^−1^, 100 μL), white light (200 mW cm^−2^, 20 min).

Motivated by the excellent performance *in vitro*, **PS1** NPs were further evaluated *in vivo* with U14 tumor-bearing mice. In Fig. 4A, obvious fluorescent signals were obtained from the site injected with **PS1** NPs, indicating good *in vivo* imaging. With increasing time, the fluorescence intensity gradually decreased, but still retained 42% of the initial intensity after 14 days (Fig. 4B), indicating the excellent *in vivo* long-term tracing ability of **PS1** NPs. After PDT treatment, faint fluorescence could be observed, suggesting high PDT efficacy of **PS1** NPs. Simultaneously, in Fig. 4C, in the control groups, the relative tumor volumes increased by 10–13 times after 14 days, implying that treatment only by irradiation or by **PS1** NPs, has negligible influence on tumor growth. As expected, tumor volume in the experimental group showed a significant reduction, confirming the good anticancer effect of **PS1** NPs under white-light irradiation. Negligible changes of body weight were observed after various treatments (ESI, Fig. S34D†) indicating the low systemic toxicity of **PS1** NPs. The biosafety of **PS1** NPs was further evaluated by hematoxylin and eosin (H&E) staining of major organs. No pathological changes were found in the major organs of the four groups. These results reveal that **PS1** NPs are excellent candidates for *in vivo* long-term tracing and image-guided PDT.

## Conclusions

In summary, we have successfully constructed for the first time a single PS to simultaneously achieve image-guided PDT, ultrafast staining and long-term tracing. Two red-emitting AIE-active D–A organic molecules and their NPs, were rationally designed and synthesized. The small Δ*E*_ST_ increases the intersystem crossing (ISC) process, leading to effective ^1^O_2_ generation for the two PSs. The construction of nanoparticles further improves the fluorescence intensity and the ^1^O_2_ production ability. Especially the **PS1** NPs are ideal for biological applications owing to the following advantages: bright red emission, large Stokes shift, high fluorescence quantum yield (40%), appropriate ^1^O_2_ generation ability, good biocompatibility, and excellent image-guided PDT activity. More importantly, **PS1** NPs can stain cells in only 5 s at room temperature, and they display excellent long-term imaging for more than 14 days *in vitro* and *in vivo*. This work successfully achieves a smart AIE PS with image-guided PDT, ultrafast staining and long-term tracing functions. This prototype example of “all-in-one” PS NPs should stimulate the design and development of new multifunctional PSs for potential clinical applications.

## Ethical statement

All animal studies were performed in strict accordance with the NIH guidelines for the care and use of laboratory animals (NIH Publication No. 85-23 Rev. 1985) and were approved by the guidelines of the Committee on Animal Use and Care of the Chinese Academy of Sciences.

## Conflicts of interest

There are no conflicts to declare.

## Supplementary Material

SC-011-C9SC06310B-s001
