# Supplementary material for: Bright red aggregation-induced emission nanoparticles for multifunctional applications in cancer therapy
Source: Chem Sci. 2020 Jan 29;11(9):2369–74. doi: 10.1039/c9sc06310b (PMC8157307; doi:10.1039/c9sc06310b)
Supplement: SC-011-C9SC06310B-s001 [file SC-011-C9SC06310B-s001.pdf]

Electronic Supplementary Material (ESI) for Chemical Science.
This journal is © The Royal Society of Chemistry 2020

Electronic supplementary information

Bright Red Aggregation-Induced Emission Nanoparticles for Multifunctional Applications in Cancer Therapy

Liping Zhang,[a] Weilong Che,[a] Zhiyu Yang,[b] Xingman Liu,[a] Shi Liu,[b] Zhigang Xie,*[b] Dongxia Zhu,*[a] Zhongmin Su,[a] Ben Zhong Tang[c]* and Martin R. Bryce*[d]

[a] *Key Laboratory of Nanobiosensing and Nanobioanalysis at Universities of Jilin Province, Department of Chemistry, Northeast Normal University, 5268 Renmin Street, Changchun, Jilin Province 130024, P. R. China*
E-mail: zhudx047@nenu.edu.cn; zmsu@nenu.edu.cn
[b] *State Key Laboratory of Polymer Physics and Chemistry, Changchun Institute of Applied Chemistry Chinese Academy of Sciences, Changchun 130022, P. R. China, E-mail: xiez@ciac.ac.cn*
[c] *State Key Laboratory of Molecular Neuroscience Institute for Advanced Study Institute of Molecular Functional Materials， The Hong Kong University of Science and Technology, Clear Water Bay, Kowloon, Hong Kong, China*
E-mail: tangbenz@ust.hk
[d] *Department of Chemistry, Durham University, Durham DH1 3LE, UK*
E-mail: m.r.bryce@durham.ac.uk

Table of Contents

1. General information and methods.. S-2

2. General information and methods.. S-6

3. Supplementary Figures and Tables S-9

1. General information and methods

Materials

Materials obtained from commercial suppliers were used without further purification unless otherwise stated. All glassware, syringes, magnetic stirring bars and needles were thoroughly dried in a convection oven. All other materials for organic synthesis were purchased from Energy Chemical Company. 1,2-Distearoyl-*sn*-glycero-3-phosphoethanolamine-*N*-[maleimide(polyethylene glycol)-2000](DSPE-PEG-Mal) was provided by Shanghai Ponsure Biotech, Inc. HIV-1 transactivator of transcription (Tat) protein-derived cell penetrating peptide (C-terminus with cysteine) was purchased from GL Biochem Co., Ltd. (Shanghai, China). Indocyanine green (ICG) was purchased from Fisher Scientific. Dulbecco's Modified Eagle Medium (DMEM), RPMI-1640 Medium, and fetal bovine serum (FBS) were purchased from Sigma-Aldrich. 2’,7’-Dichlorofluorescence diacetate (DCFH-DA) were purchased from Shanghai Beyotime Biotechnology Co., Ltd.. 3-(4,5-Dimethylthiazol-2-yl)-2,5-diphenyltetrazolium bromide (MTT) was obtained from Shanghai Beyotime Biotechnology Co., Ltd. (China). The cell viability (live dead cell staining) assay kit was purchased from Jiangsu KeyGEN Biotechnology Co., Ltd. (China). Milli-Q water was collected from a Milli-Q system (Millipore).

Measurements

Reactions were monitored using thin layer chromatography (TLC). ^{1}H NMR spectra were recorded at 25 ^{o}C on a Varian 500 MHz spectrometer. The chemical shifts (δ) are given in parts per million relative to internal standard TMS. The ^{1}H NMR spectra were referenced internally to the residual proton resonance in $CDCl_3$ (δ 7.24 ppm). The mass spectra (MS) of the samples were obtained on a Bruker autoflex III smart beam mass spectrometer (MALDI-TOF/TOF) with a smart beam laser at 355 nm wavelength. Elemental analysis was performed on a FlashEA1112 analyzer. UV–vis absorption spectra were monitored with a Shimadzu UV-2450 PC UV–vis spectrophotometer. The fluorescence spectra, excited state lifetimes (τ) and fluorescence quantum yields (Φ_{FL}) were recorded on an Edinburgh FLS920 spectrofluorimeter under air at room temperature. Size and size distribution (DLS) of the nanoparticles were characterized by Malvern Zeta-sizer Nano. Transmission electron microscopy (TEM) images of the samples were taken by a TECNAI F20 microscope. Confocal lasing scanning microscopic (CLSM) images were obtained on a Zeiss Laser Scanning Confocal Microscope; LSM710. The mouse was imaged by using a Maestro *in vivo* optical imaging system (Cambridge Research & Instrumentation, Inc., Woburn, Massachusetts).

Density functional theory calculations

All the calculations were performed in the gas phase using a Gaussian 09 program.[1] The ground-state structure was optimized with B3LYP method and 6-311G(d, p) basis set. Then, a vertical excitation was carried out based on the optimized structure with the same method, from which the ground-state molecular orbital energy was obtained.

Preparation of AIE nanoparticles

AIE molecules (1 mg) and 1,2-Distearoyl-*sn*-glycero-3-phosphoethanolamine-*N*-[maleimide(polyethylene glycol)-2000](DSPE-PEG-Mal) (2 mg) were dissolved in THF 1 mL), and then poured into Milli-Q water (10 mL), followed with sonication for 2 min with a microtip probe sonicator (XL2000, Misonix Incorporated, NY). The residue THF solvent was evaporated by violent stirring the

suspension overnight. Subsequently, HIV-1 Tat (500 μg/mL, 150 μL) was added into the above solution and stirred at room temperature for 12 h. The excess HIV-1 Tat was removed by dialysis using a 10 000 molecular weight cutoff membrane. NPs were obtained by filtration through a 0.2 μm syringe-driven filter.

Cell culture

HeLa cells (the human cervical cancer cell line) and H22 cells (the murine hepatic carcinoma cell line) were purchased from Jilin University and grown in Dulbecco's modified Eagle's medium (DMEM, GIBCO) supplemented with 10% heat-inactivated fetal bovine serum (FBS, GIBCO), 100 U mL penicillin and 100 μg mL streptomycin (Sigma). The cells were maintained in a humidified incubator at 37 ^{o}C with 5% CO_2.

Cytotoxicity assay

HeLa cells were harvested in a logarithmic growth phase and seeded in 96-well plates at a density of 10^4 cells per well for 24 h. Then different concentrations of **PS1**/**PS2** and **PS1**/**PS2** NPs of various concentrations (0-20 μg mL^{-1}) were added into cell culture medium separately. After incubating for 6 h, the light group received white irradiation (20 mW cm^{-2}) for 60 min, then continued incubation to 24 h. The dark control group received nothing and were incubated for the same time as the control group. Subsequently the MTT (20 μL) was added to the medium for 4 h. The absorbance of MTT was measured by a Bio-Rad 680 microplate reader at 490 nm.

Intracellular ROS assays

HeLa cells were seeded in 6-well culture plates at a density of 5×10^4 cells per well for 24 h. Then **PS2** and **PS2** NPs (10 μg mL^{-1}) were added into the cell culture medium separately. After incubating for 6 h, the light group received irradiation (20 mW cm^{-2}) for 20 min, whereas the dark control group received nothing. Then using the DMEM solution without FBS the cells were washed. The DMEM solution containing DCFH-DA (10^{-5} mol L^{-1}) was added and incubated for 30 min. The cells were observed as soon as possible by CLSM with excitation at 488 nm.

Live/ dead cell staining assays

HeLa cells were harvested in a logarithmic growth phase and seeded in 96-well plates at a density of 10^4 cells per well for 24 h. The **PS1/PS2 and PS1/PS2** NPs (20 μg mL^{-1}) were added into cell culture medium separately. After incubating for 6 h, the light group received white irradiation (20 mW cm^{-2}) for 60 min, then incubation was continued to 24 h. The dark control group received nothing and was incubated for the same time as the control. After staining with calcein-AM/PI for 40 min, phosphate-buffered saline (PBS) was used to wash the cells. Finally the cells were observed by Nikon C1 Si laser scanning confocal microscopy.

Cellular Uptake

The cellular uptake measurement was investigated by CLSM. Cells harvested in a logarithmic growth phase were seeded in 6-well plates at a density of 5×10^4 cells/well and incubated in DMEM for 24 h. The medium was then replaced by 2 mL of DMEM containing AIE NPs (0.5 μg mL^{-1} or 2 μg mL^{-1}or 2 μg mL^{-1} or 3 μg mL^{-1}) and incubated for different times at 37 °C and further washed 3 times with PBS buffer. For the CLSM, the cells were fixed with 4% paraformaldehyde solution for 10 min. Then, the cells were washed with PBS and observed using confocal laser scanning microscopy with excitation at 488 nm (CLSM, Zeiss LSM 700).

Long-Term Cellular Tracing

Cells harvested in a logarithmic growth phase were seeded in 6-well plates at a density of 5×10^4 cells/well and incubated in DMEM for 24 h. The medium was then replaced by 2 mL of DMEM containing 20 μg mL^{-1} of AIE NPs and incubated for 6 h at 37 °C (day 0). Then the cells were diluted and subcultured in 6-well plates for 0 to 15 days regeneration, respectively. Upon reaching the designated day, the cells were washed with PBS buffer and then fixed with 4% of paraformaldehyde solution for 10 min. Later, the cells were washed with PBS and observed using confocal laser scanning microscopy with excitation at 488 nm (CLSM, Zeiss LSM 700).

Tumor-bearing mouse model

All animal studies were performed in strict accordance with the NIH guidelines for the care and use of laboratory animals (NIH Publication No. 85-23 Rev. 1985) and were approved by the guidelines of the Committee on Animal Use and Care of the Chinese Academy of Sciences. Male mice were purchased from Jilin University, China (6 weeks, 15–20 g). Inoculating the subcutaneous murine U14 cells (100 μL) into the right thigh established the tumor-bearing mouse model.

In Vivo **Imaging**

Time-dependent *in vivo* fluorescence images: The mice with tumors were intratumoral injected with **PS1** NPs (100 μg mL^{-1}, 100 μL) or **PS1** NPs (100 μg mL^{-1}, 100 μL) + white irradiation (200 mW cm^{-2}, 20 min). Then, under anesthesia, the *in vivo* imaging was performed using an *in vivo* imaging system (excitation, 500- 565 nm; emission, 580-750 nm). Maestro software was used to remove the mouse background fluorescence.

In vivo **PDT**

After one week, the mice were intratumoral injected with **PS1** NPs (100 μg mL^{-1}, 100 μL), the control group received saline instead. After 1 h the tumor site of the mouse was subjected to laser irradiation (200 mW cm^{-2}) for 20 min. The tumor volume and body weight of the mice were measured every 2 days for 14 days. The tumor size was measured every other day and calculated as follows: volume = (tumor length) × $(\text{tumor width})^2/2$.

Histological analysis

The mice were sacrificed at day 14, and major organs and the tumors were collected and fixed in 4% paraformaldehyde. Then they were embedded into paraffin, and sliced at a thickness of 5 μm. Slices were stained with hematoxylin and eosin (H&E) and imaged by optical microscopy.

2. General information and methods

Synthesis of S1

4-(Diphenylamino)phenylboronic acid (0.694 g, 2.4 mmol), 4-bromobenzophenone (0.522 g, 2.0 mmol), $Pd(PPh_3)_4$ (0.058g, 0.05 mmol) and sodium carbonate solution (2 M, 10 mL) was dissolved in THF (40 mL) and CH_3OH (10 mL) was stirred at 80 °C for 24 h under a nitrogen atmosphere. After cooling down the reaction mixture to ambient temperature, it was extracted with dichloromethane and washed with water. The dichloromethane layer was separated and dried over $MgSO_4$. After solvent evaporation, the crude product was purified by column chromatography on silica gel using *n*-hexane/dichloromethane (1/1, v/v) as the eluent to afford **S1** as a yellow liquid (0.646 g, 76% yield). ^1H NMR (500 MHz, $CDCl_3$, δ [ppm]): 7.88 (d, J = 9.5 Hz, 2H, ArH), 7.82 (d, J = 6.0 Hz, 2H, ArH), 7.67 (d, J = 7.0 Hz, 2H, ArH), 7.59 (t, J = 4.5 Hz, 1H, ArH), 7.53-7.49 (m, 4H, ArH),7.28 (t, J = 6.5 Hz, 4H, ArH), 7.15 (d, J = 7.5 Hz, 6H, ArH), 7.07 (d, J = 6.5 Hz, 2H, ArH).

Synthesis of S2

4-(Diphenylamino)phenylboronic acid (0.694 g, 2.4 mmol), 4,4'-dibromobenzophenone (0.340 g, 1.0 mmol), $Pd(PPh_3)_4$ (0.058g, 0.05 mmol) and sodium carbonate solution (2 M, 10 mL) was dissolved in THF (40 mL) and CH_3OH (10 mL) was stirred at 80 °C for 24 h under a nitrogen atmosphere. After cooling down the reaction mixture to ambient temperature, it was extracted with dichloromethane and washed with water. The dichloromethane layer was separated and dried over $MgSO_4$. After solvent evaporation, the crude product was purified by column chromatography on silica gel using *n*-hexane/dichloromethane (1/1, v/v) as the eluent to afford **S2** as a yellow liquid (0.463 g, 69% yield). ^1H NMR (500 MHz, $CDCl_3$, δ [ppm]): 7.90 (d, J = 6.5 Hz, 4H, ArH), 7.869 (d, J = 7.0 Hz, 4H, ArH), 7.54 (d, J = 8.5 Hz, 4H, ArH), 7.29 (t, J = 4.5 Hz, 8H, ArH), 7.15 (d, J = 6.0 Hz, 12H, ArH),7.06 (t, J = 6.0 Hz, 4H, ArH).

Synthesis of PS1

To the solution of compound **S1** (0.425 g, 1.0 mmol) and malononitrile (0.198 g, 3.0 mmol) in dichloromethane (60 mL) was added titanium tetrachloride (0.4 mL, 3.5 mmol) slowly at 0 °C. After the reaction mixture was stirred for 30 min, pyridine (0.3 mL, 3.5 mmol) was injected and stirred for

another 30 min. Then the mixture was heated at 40 °C for 4 h. After the mixture was cooled down to room temperature, the reaction was quenched by water (300 mL) and the mixture was extracted with dichloromethane. The collected organic layer was washed with brine, dried over Na_2SO_4 and concentrated under reduced pressure. After solvent evaporation, the crude product was purified by column chromatography on silica gel using *n*-hexane/dichloromethane (1/2, v/v) as the eluent to afford **PS1** as a red liquid (0.385 g, 81% yield). ^{1}H NMR (500 MHz, $CDCl_3$, δ [ppm]): 7.67 (d, J = 5.0 Hz, 2H, ArH), 7.59 (t, J = 5.5 Hz, 1H, ArH), 7.50 (t, J = 6.5 Hz, 6H, ArH), 7.46 (d, J = 5.0 Hz,2H, ArH), 7.29 (t, J = 5.0 Hz, 4H, ArH), 7.14 (d, J = 8.0 Hz, 6H, ArH), 7.07 (t, J = 5.0 Hz, 2H, ArH). ^{13}C NMR (151 MHz, $CDCl_3$) δ 174.40, 148.54, 147.30, 145.20, 136.26, 133.98, 132.53, 132.29, 131.28, 130.50, 129.42, 128.85, 127.88, 126.58, 124.97, 123.56, 122.99, 114.24, 114.12, 80.63. MS: (MALDI-TOF) [m/z]: 473.2 (calcd: 473.6). Anal. Calcd. for $C_{34}H_{23}N_3$: C 86.23, H 4.90, N 8.87. Found C 86.21, H 4.91, N 8.88%.

Synthesis of PS2

To the solution of compound **S2** (0.669 g, 1.0 mmol) and malononitrile (0.198 g, 3.0 mmol) in dichloromethane (60 mL) was added titanium tetrachloride (0.4 mL, 3.5 mmol) slowly at 0 °C. After the reaction mixture was stirred for 30 min, pyridine (0.3 mL, 3.5 mmol) was injected and stirred for another 30 min. Then the mixture was heated at 40 °C for 4 h. After the mixture was cooled down to room temperature, the reaction was quenched by water (300 mL) and the mixture was extracted with dichloromethane. The collected organic layer was washed with brine, dried over Na_2SO_4 and concentrated under reduced pressure. After solvent evaporation, the crude product was purified by column chromatography on silica gel using n-hexane/dichloromethane (1/2, v/v) as the eluent to afford **PS2** as a red liquid (0.535 g, 75% yield). ^{1}H NMR (500 MHz, $CDCl_3$, δ [ppm]): 7.90 (d, J = 8.0 Hz, 2H, ArH), 7.69 (t, J = 5.0 Hz, 4H, ArH), 7.54 (d, J = 7.5 Hz, 4H, ArH), 7.51 (d, J = 5.5 Hz,2H, ArH), 7.29 (t, J = 6.5 Hz, 8H, ArH), 7.15 (d, J = 5.0 Hz, 12H, ArH), 7.08-7.05 (m, 4H, ArH). ^{13}C NMR (151 MHz, $CDCl_3$) δ 148.12, 147.46, 147.32, 144.58, 135.94, 133.34, 131.38, 130.72, 129.41, 129.37, 127.94, 127.89,

126.59, 126.26, 124.95, 124.78, 123.53, 123.35, 123.33, 123.03. MS: (MALDI-TOF) [m/z]: 716.3 (calcd: 716.9). Anal. Calcd. for $C_{52}H_{36}N_4$: C 87.12, H 5.06, N 7.82. Found C 87.11, H 5.05, N 7.84%.

3. Supplementary Figures and Tables

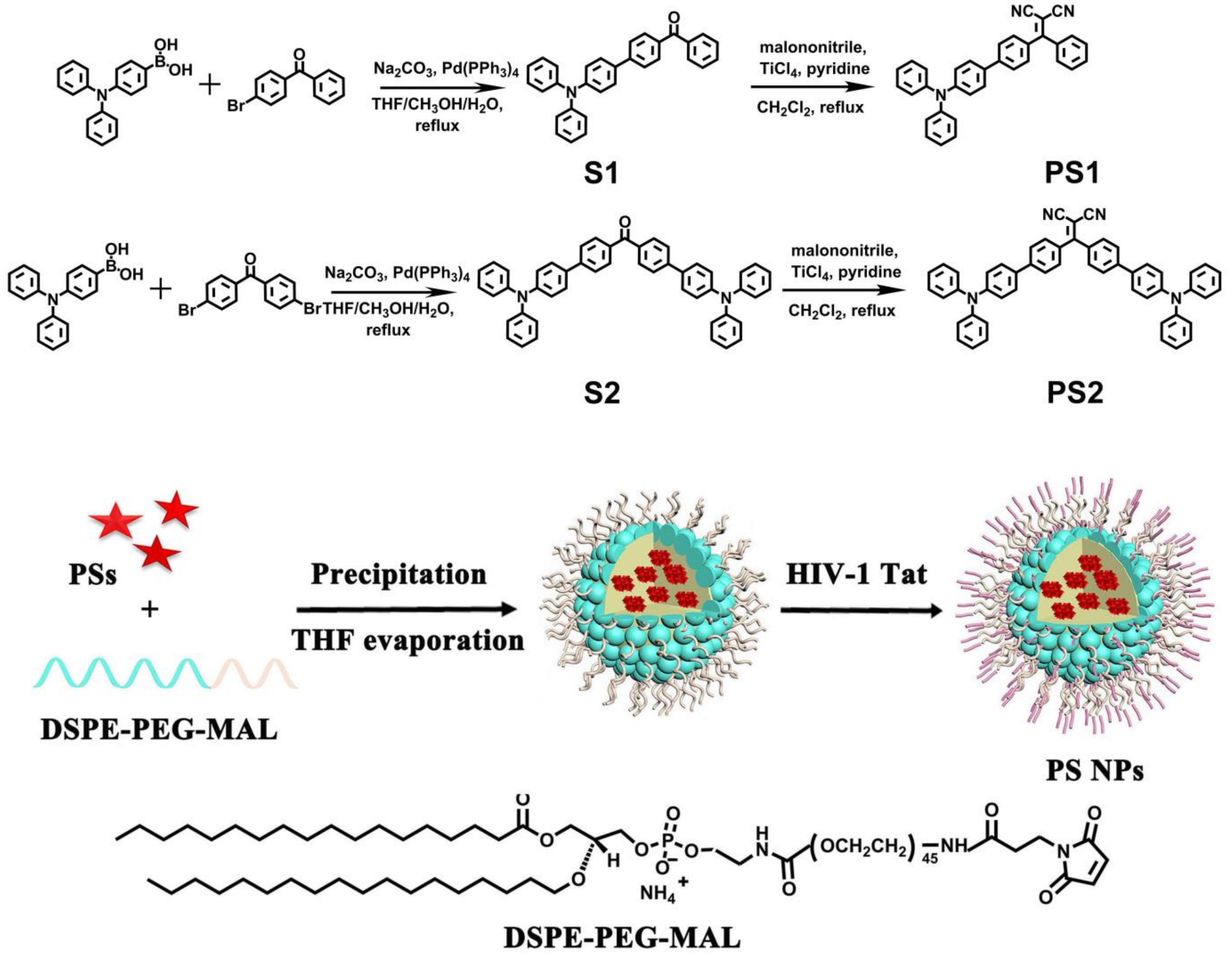

Scheme S1. Synthetic routes of **PS1**, **PS2** and AIE NPs.

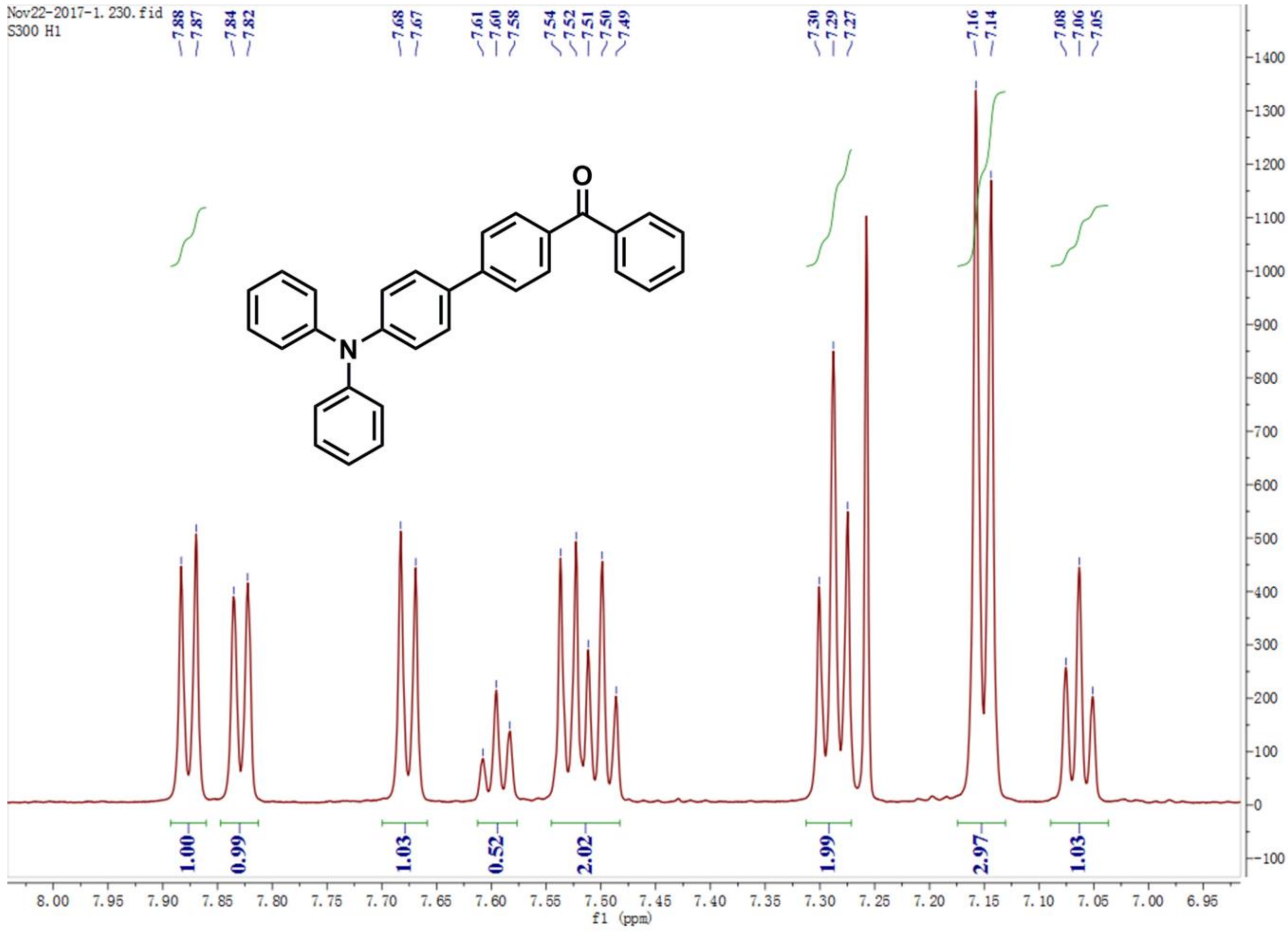

Figure S1. ^{1}H NMR spectrum of **S1** in $CDCl_3$.

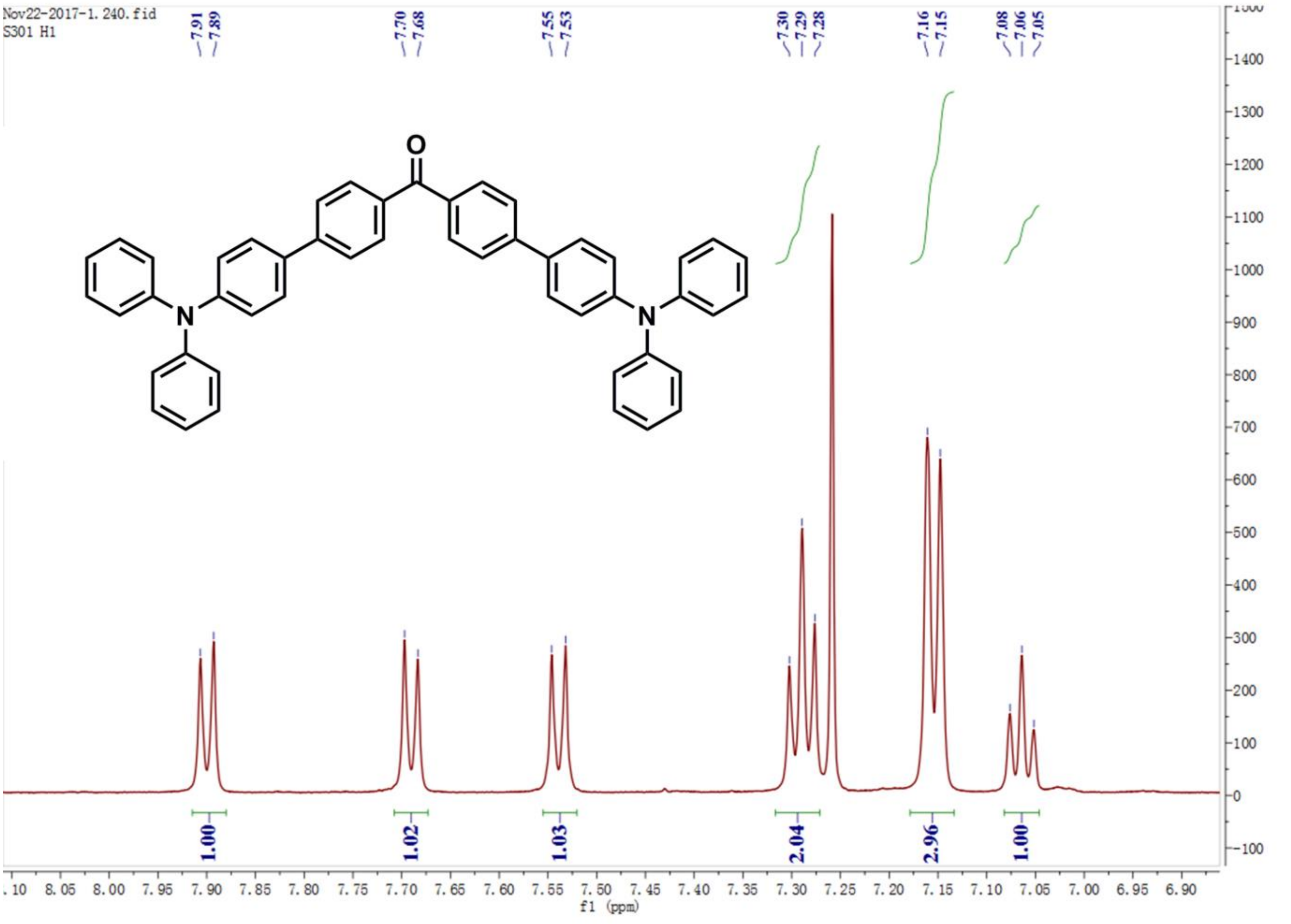

Figure S2. ^{1}H NMR spectrum of **S2** in $CDCl_3$.

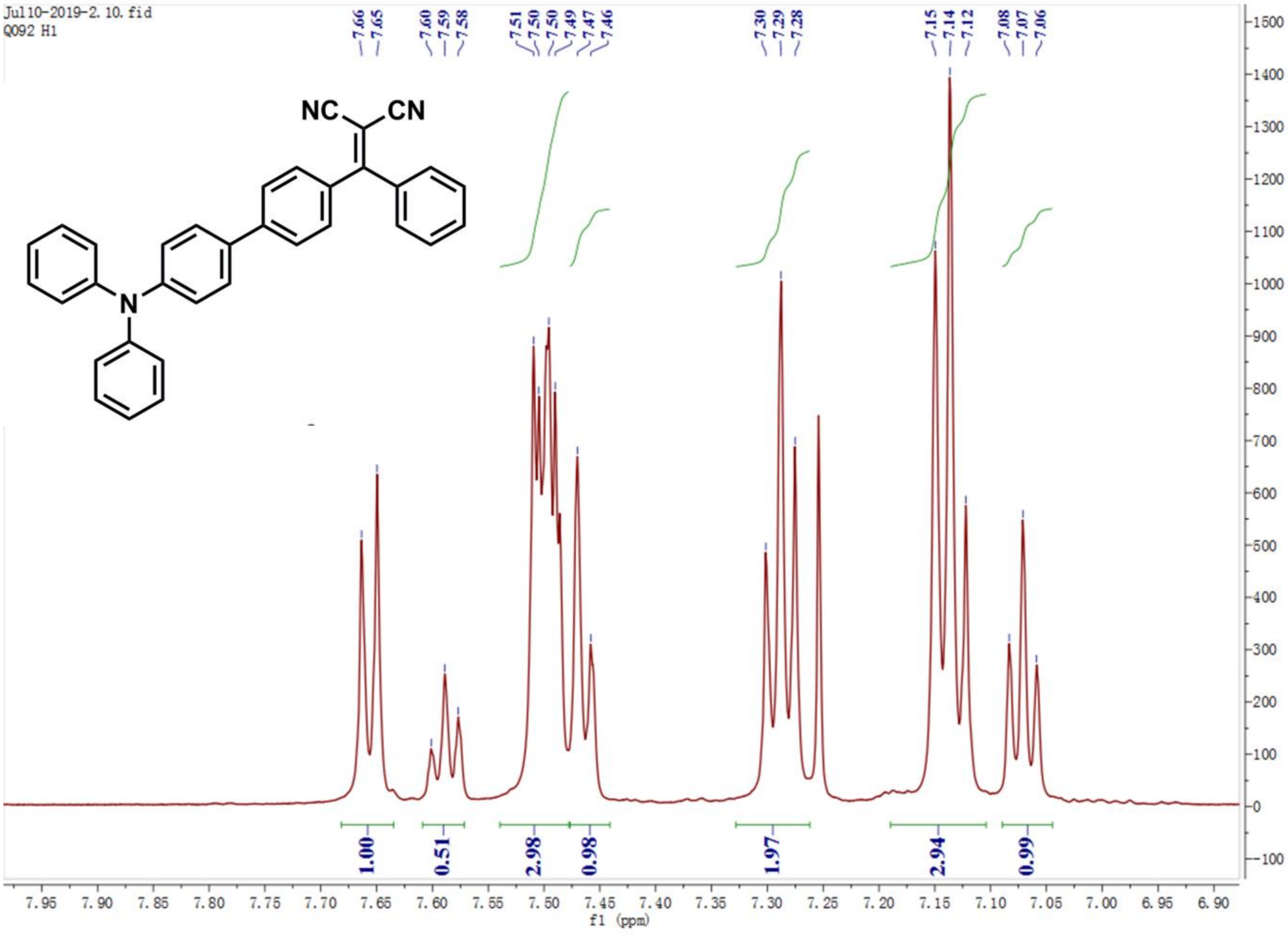

Figure S3. ^{1}H NMR spectrum of **PS1** in $CDCl_3$.

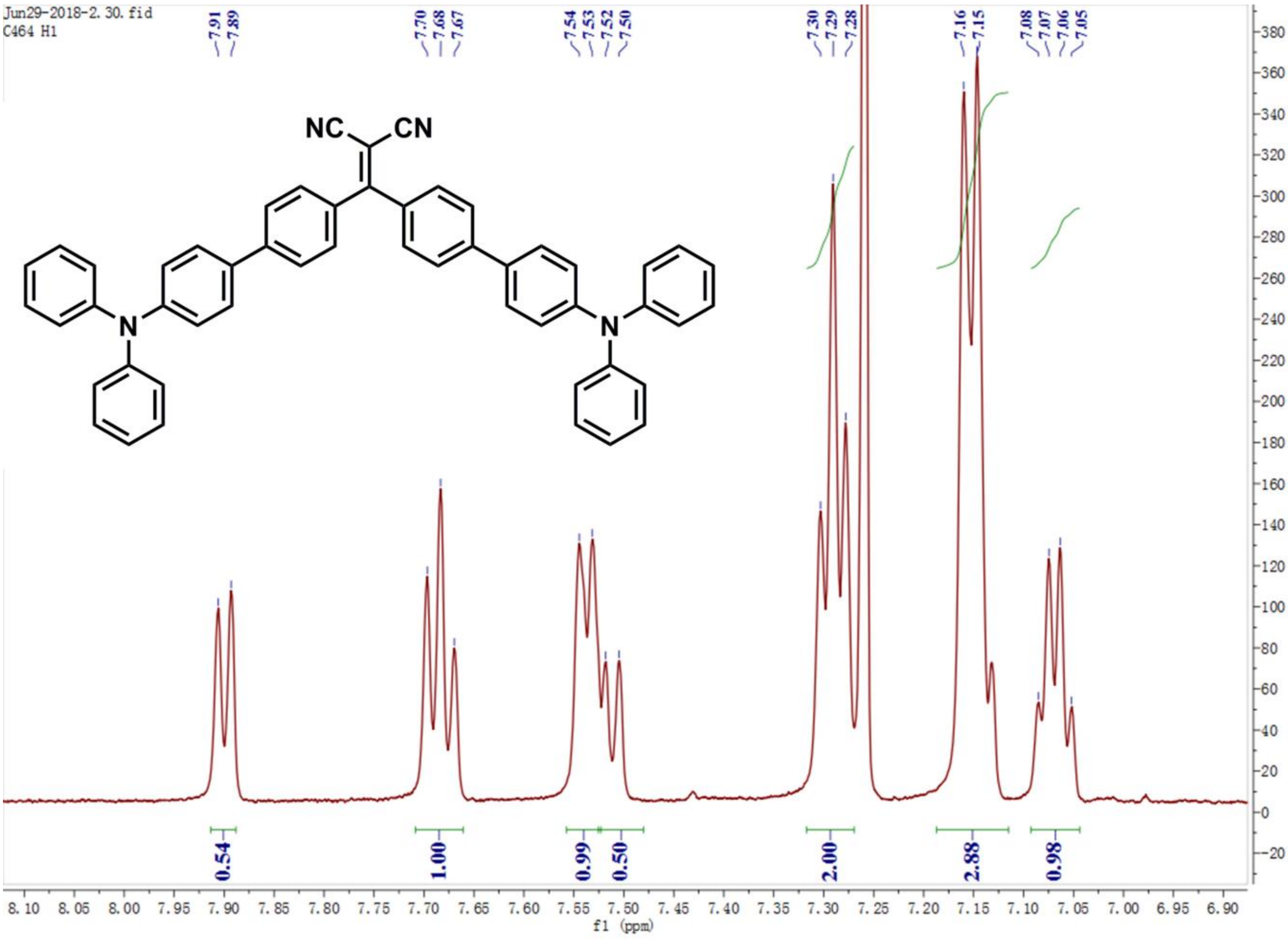

Figure S4. ^{1}H NMR spectrum of **PS2** in $CDCl_3$.

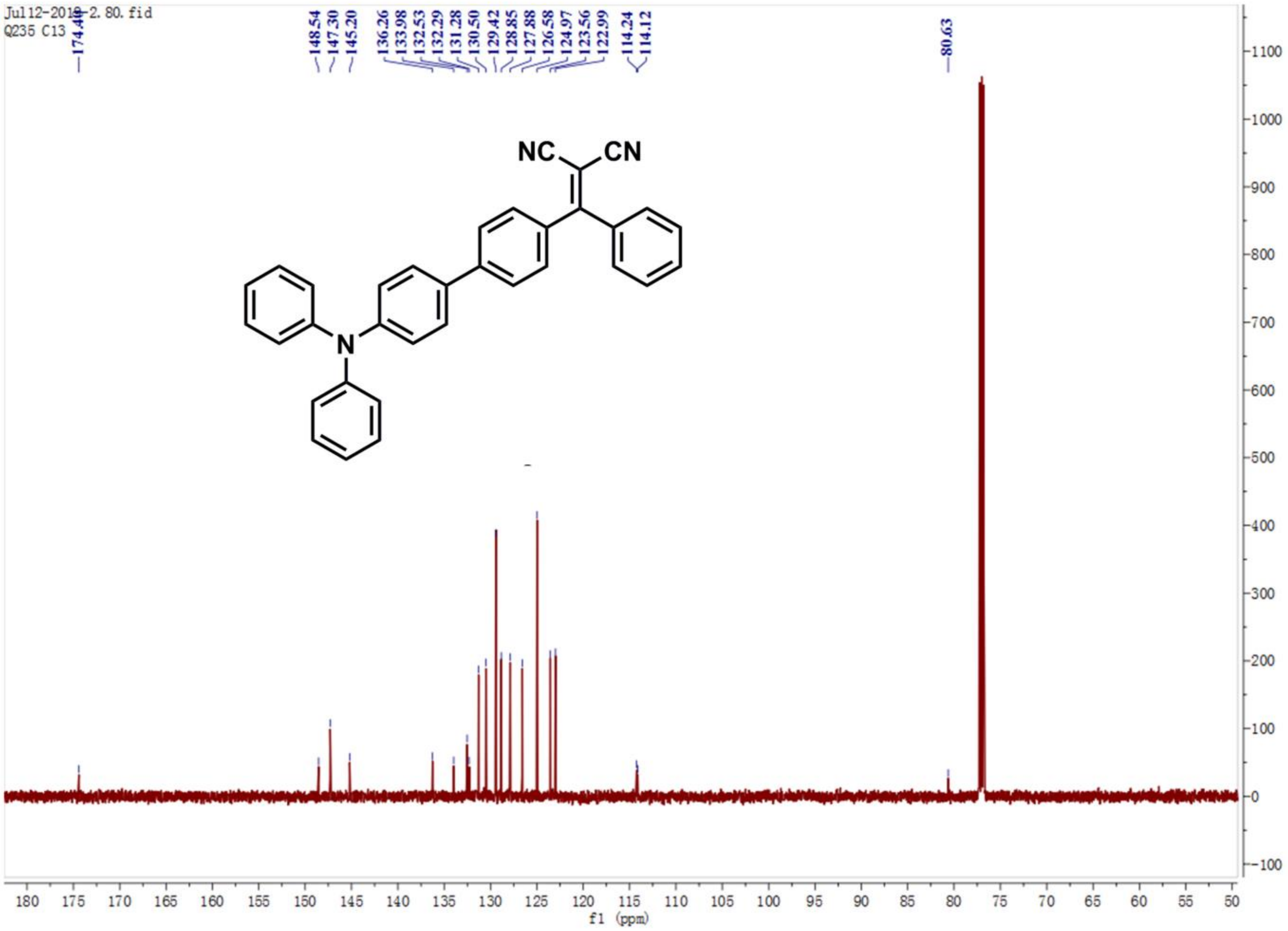

Figure S5. ^{13}C NMR spectrum of **PS1** in $CDCl_3$.

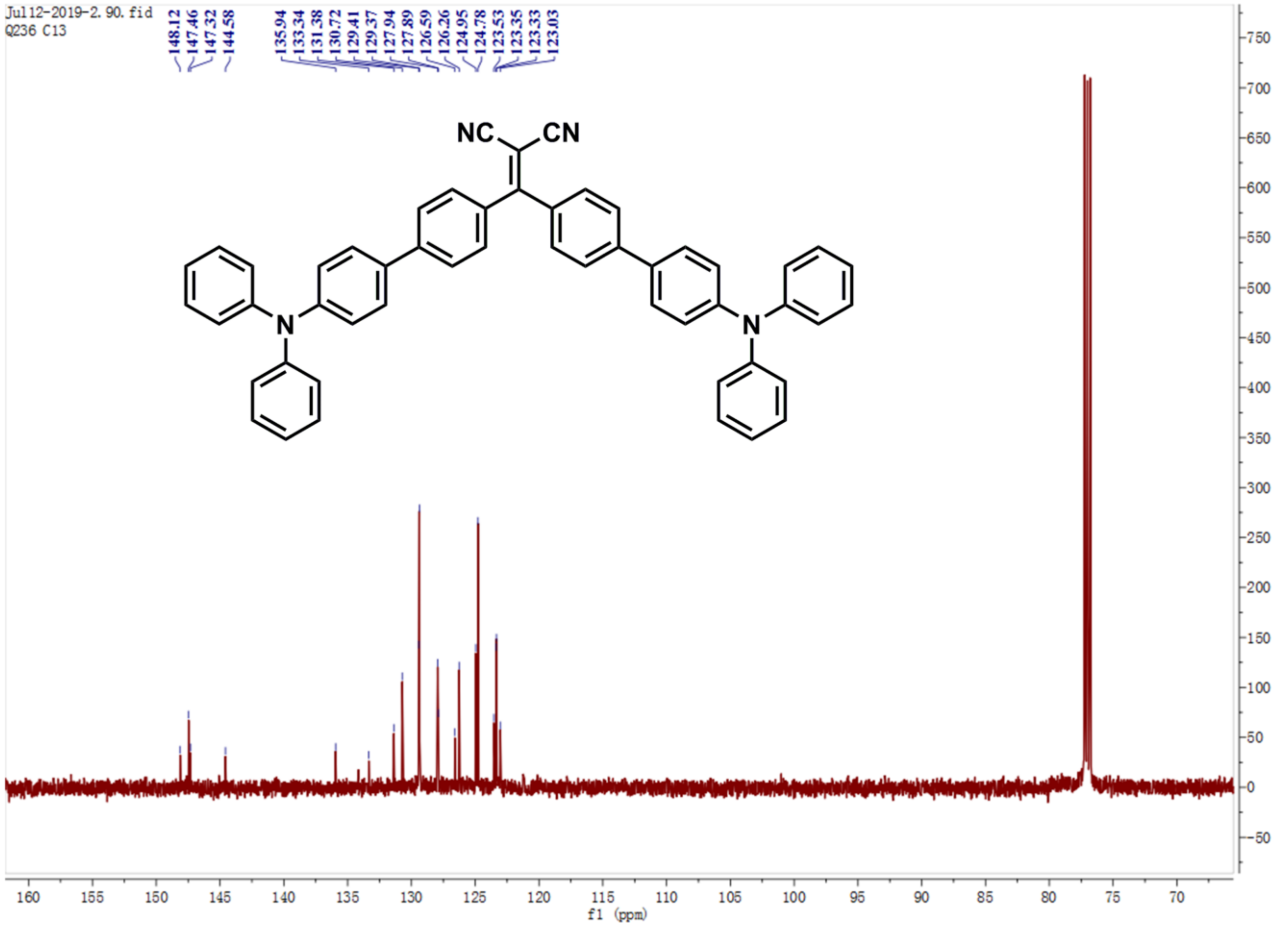

Figure S6. ^{13}C NMR spectrum of **PS2** in $CDCl_3$.

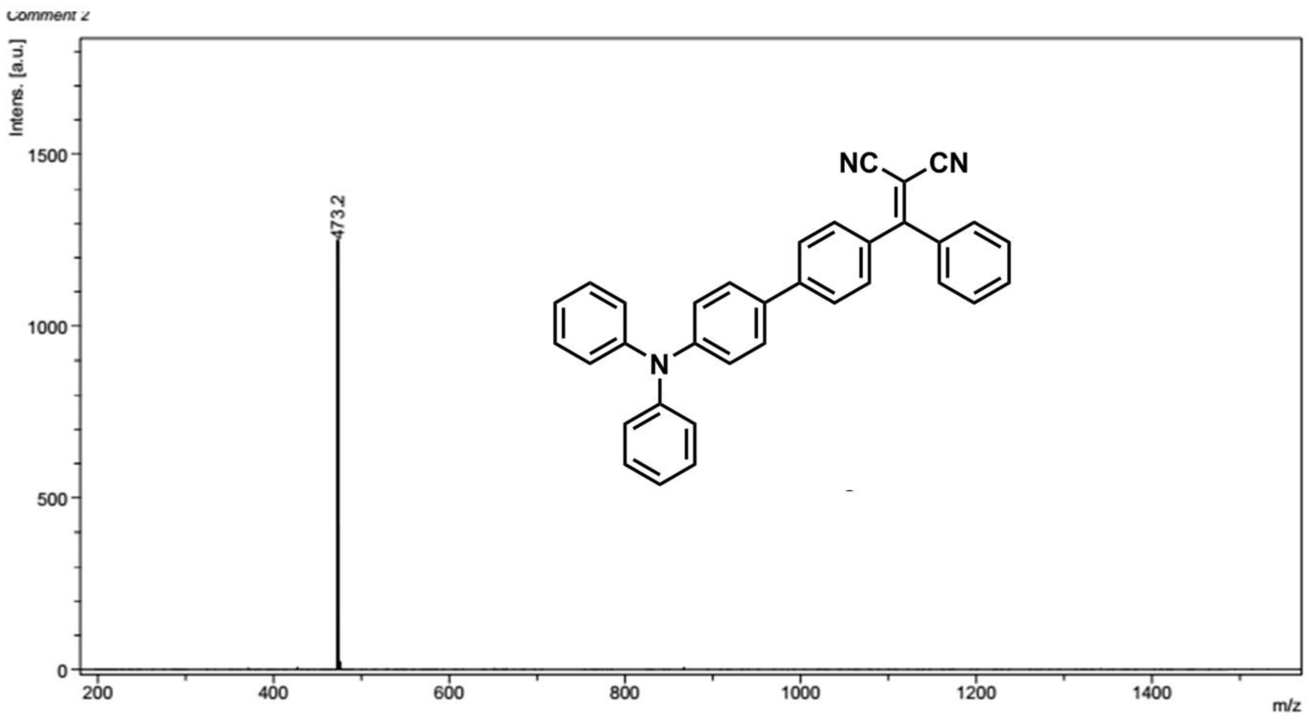

Figure S7. MALDI-TOF mass spectrum of **PS1**.

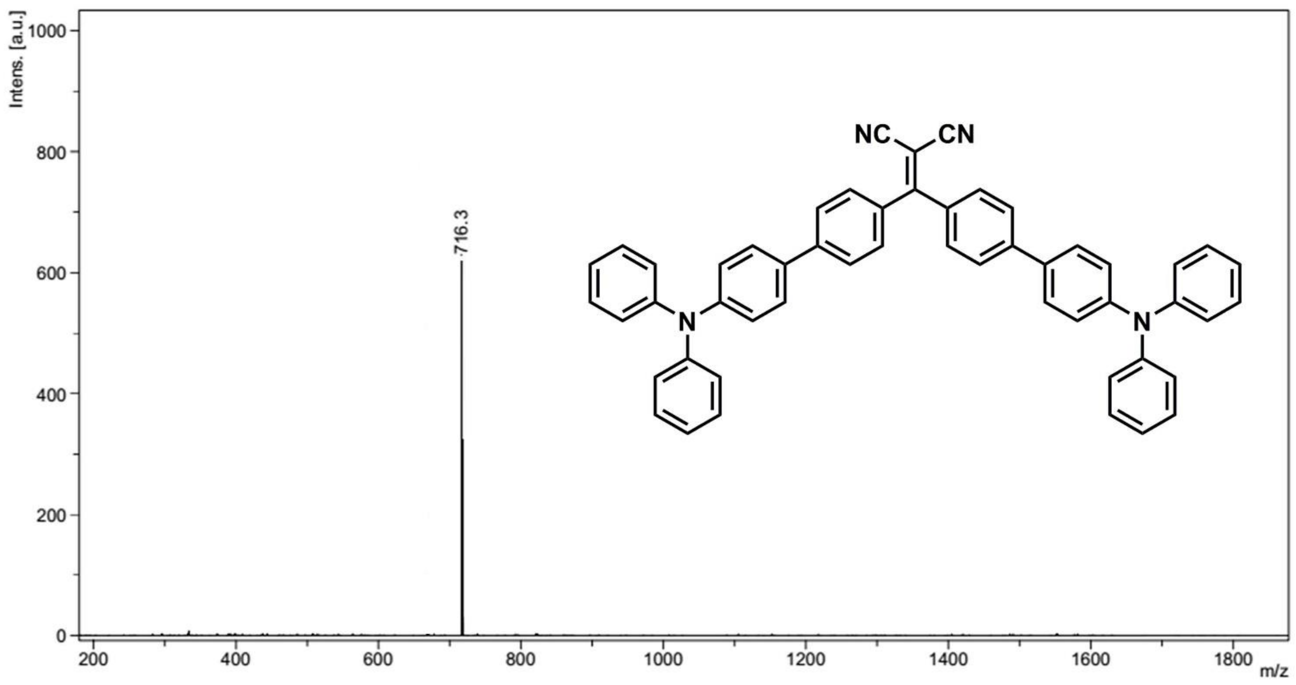

Figure S8. MALDI-TOF mass spectrum of **PS2**.

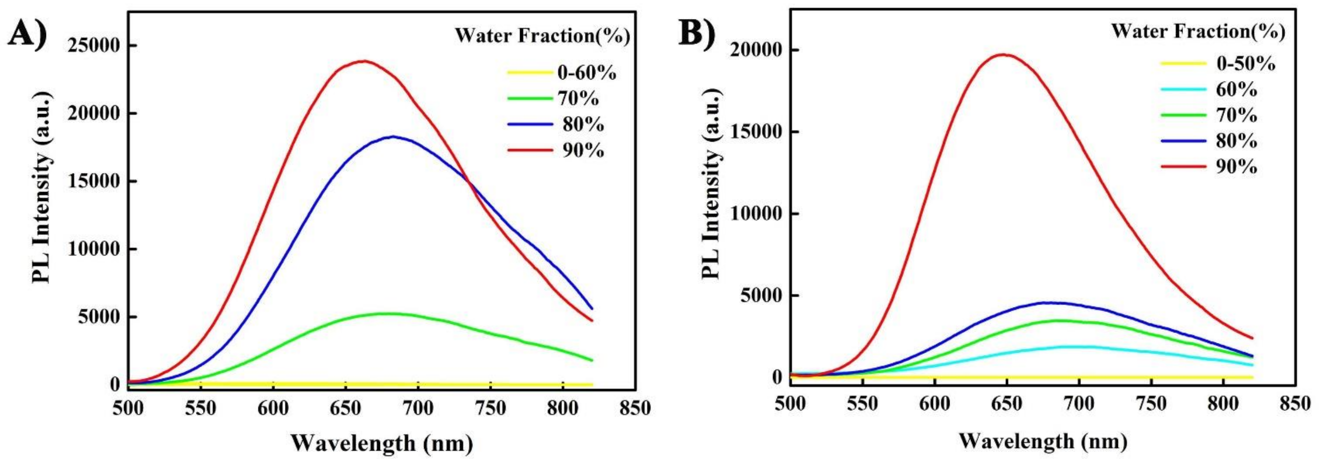

Figure S9. Emission spectra of A) **PS1** and B) **PS2** (10^{-5} M) in DMSO–water mixtures with different water fractions (0–90% v/v) at room temperature.

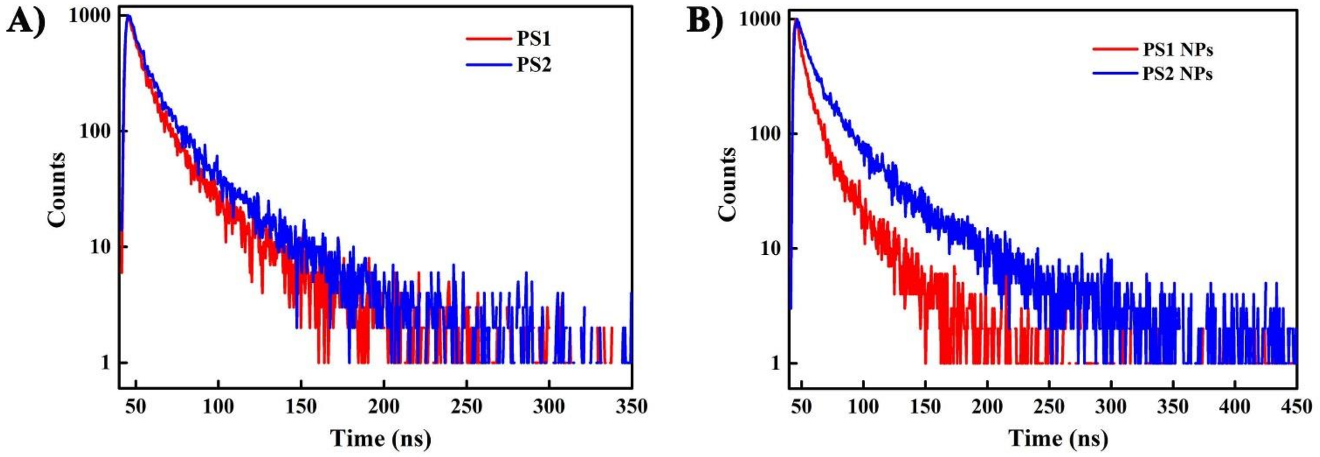

Figure S10. A) The fluorescence lifetimes of **PS1** and **PS2**. B) The fluorescence lifetimes of **PS1** NPs and **PS2** NPs.

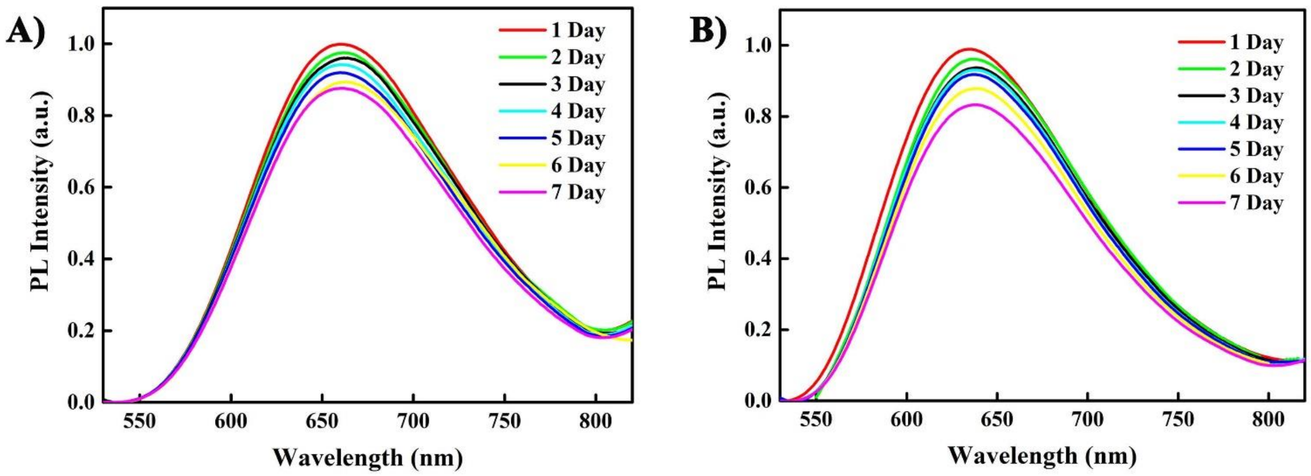

Figure S11. The fluorescence intensity of A) **PS1** NPs and B) **PS2** NPs during seven days.

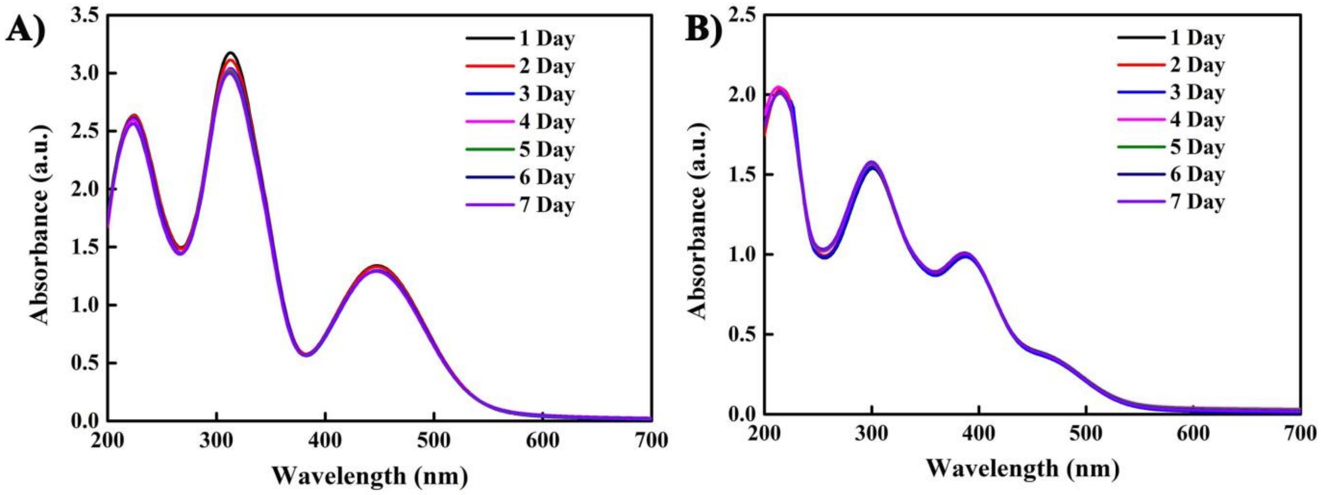

Figure S12. The absorbance intensity of A) **PS1** NPs and B) **PS2** NPs during seven days.

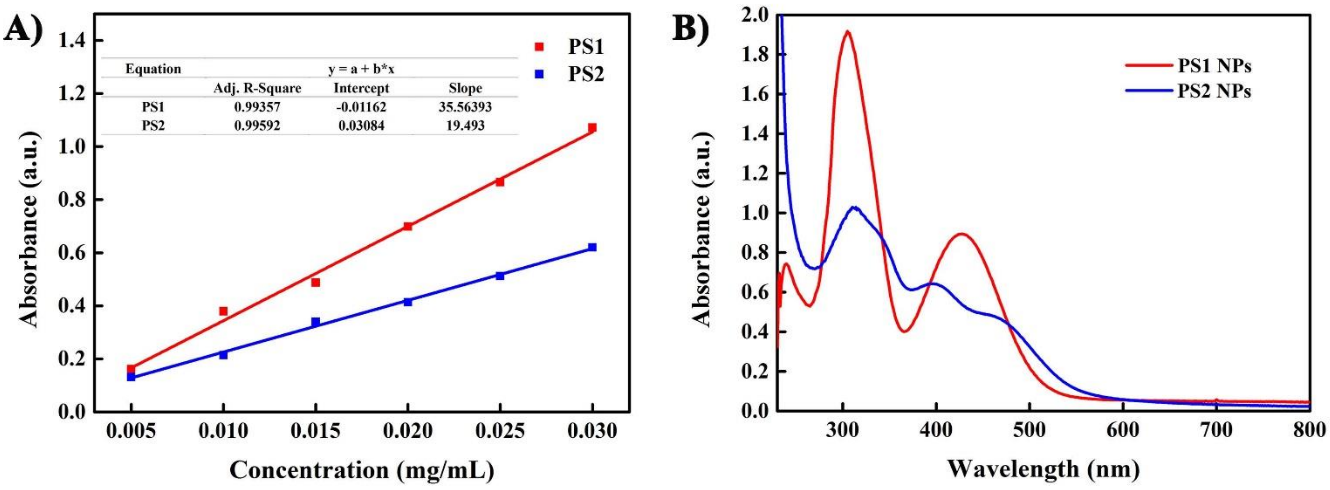

Figure S13. A) Standard curve of **PS1** and **PS2** in DMSO and water (4:1 v/v); B) the UV-visible absorption spectra of **PS1** NPs and **PS2** NPs (1 mL) in DMSO (4 mL).

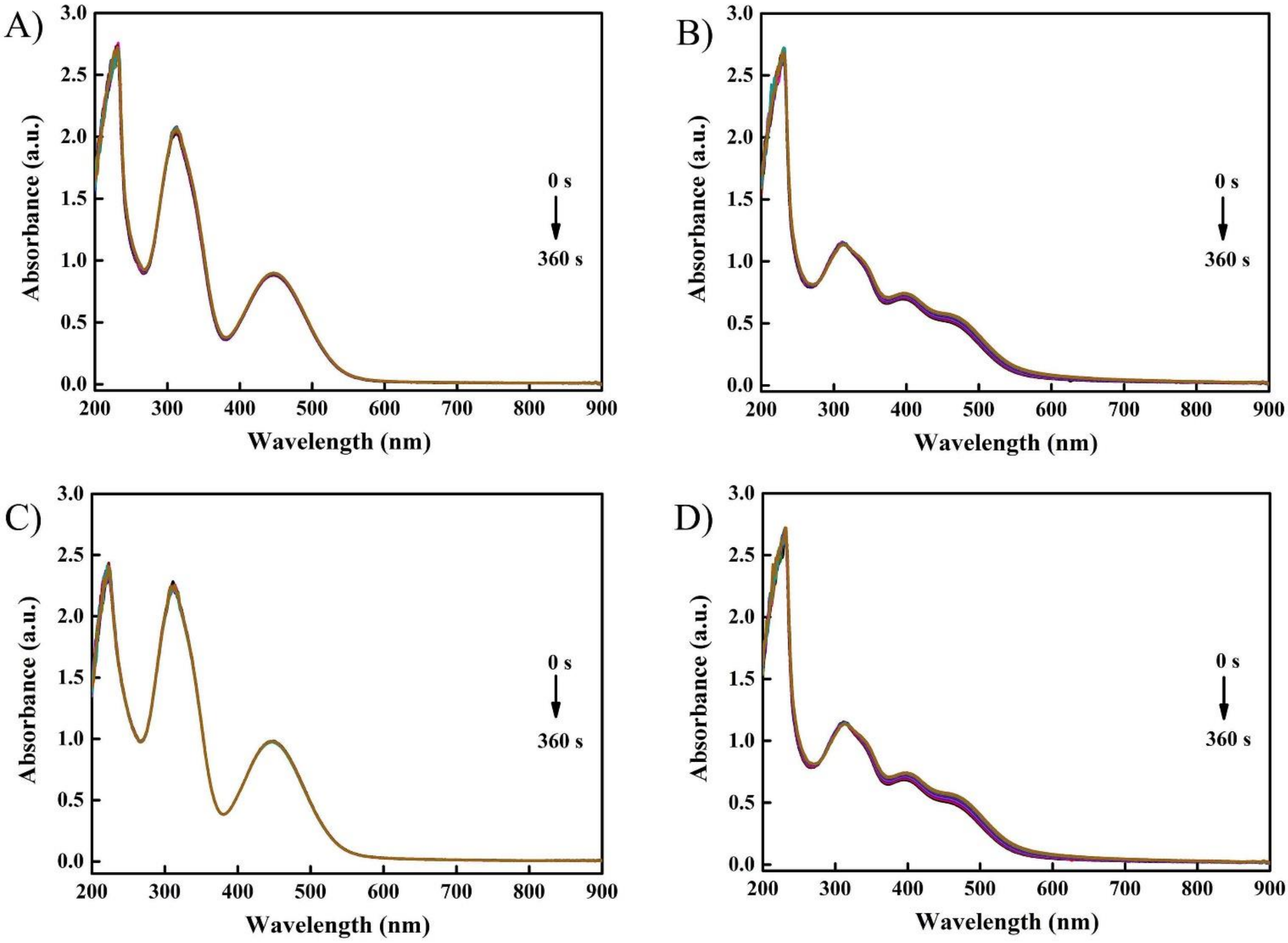

Figure S14. UV–vis absorption spectra of A) **PS1**, B) **PS2**, C) **PS1** NPs and D) **PS2** NPs (30 μg mL^{-1}) for different times under white irradiation (20 mW cm^{-2}).

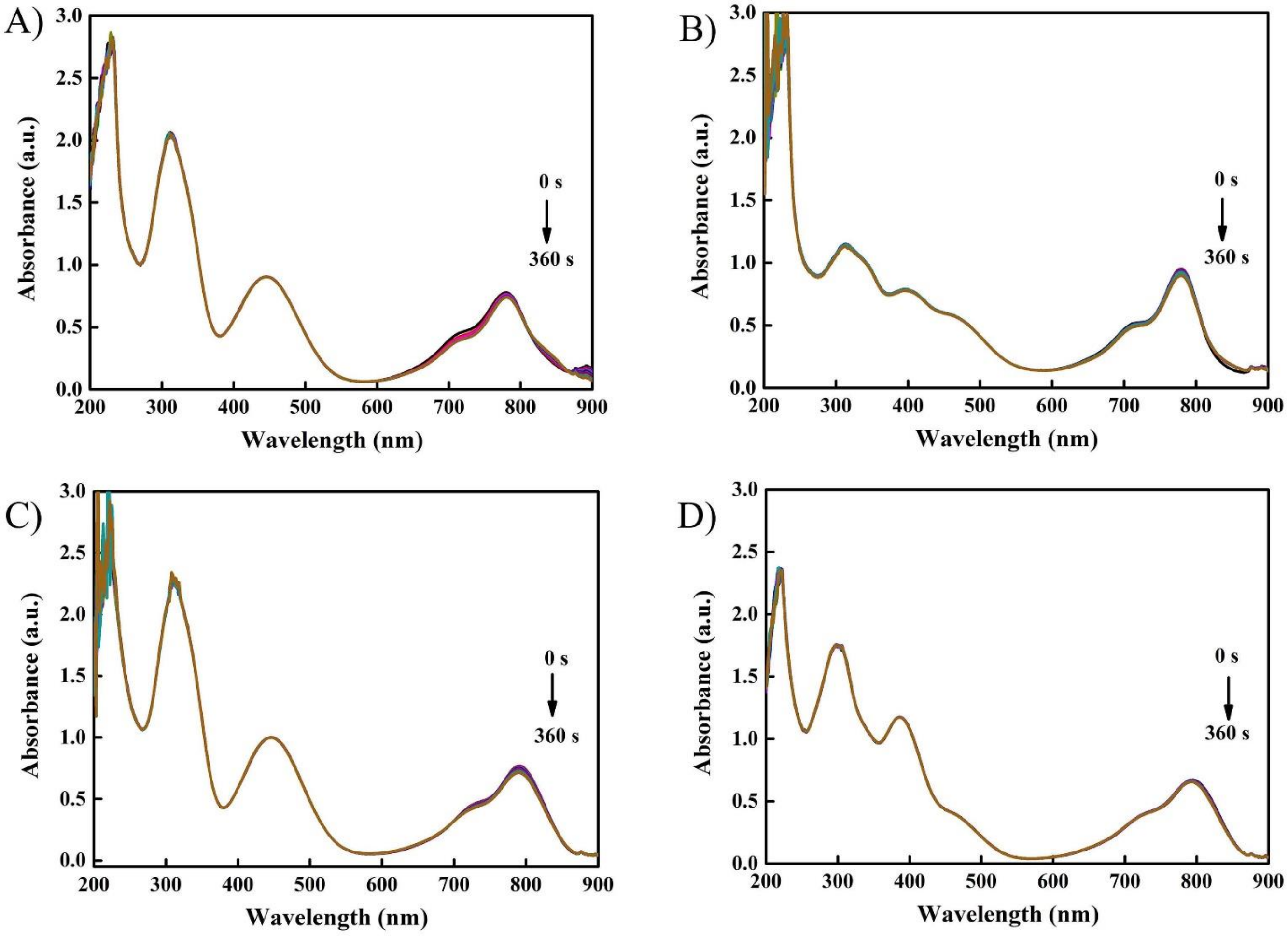

Figure S15. UV–vis absorption spectra of ICG (5 μg mL^{-1}) in the presence of A) **PS1**, B) **PS2**, C) **PS1** NPs and D) **PS2** NPs (30 μg mL^{-1}) for different times.

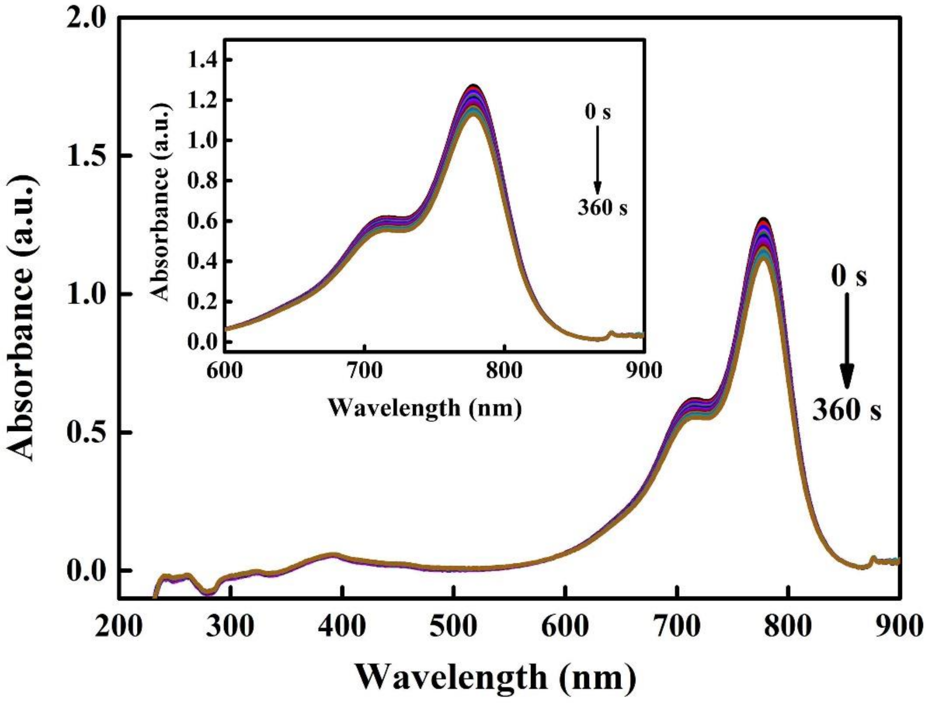

Figure S16. UV–vis absorption spectra of ICG (5 μg mL^{-1}) for different times under white irradiation (20 mW cm^{-2}).

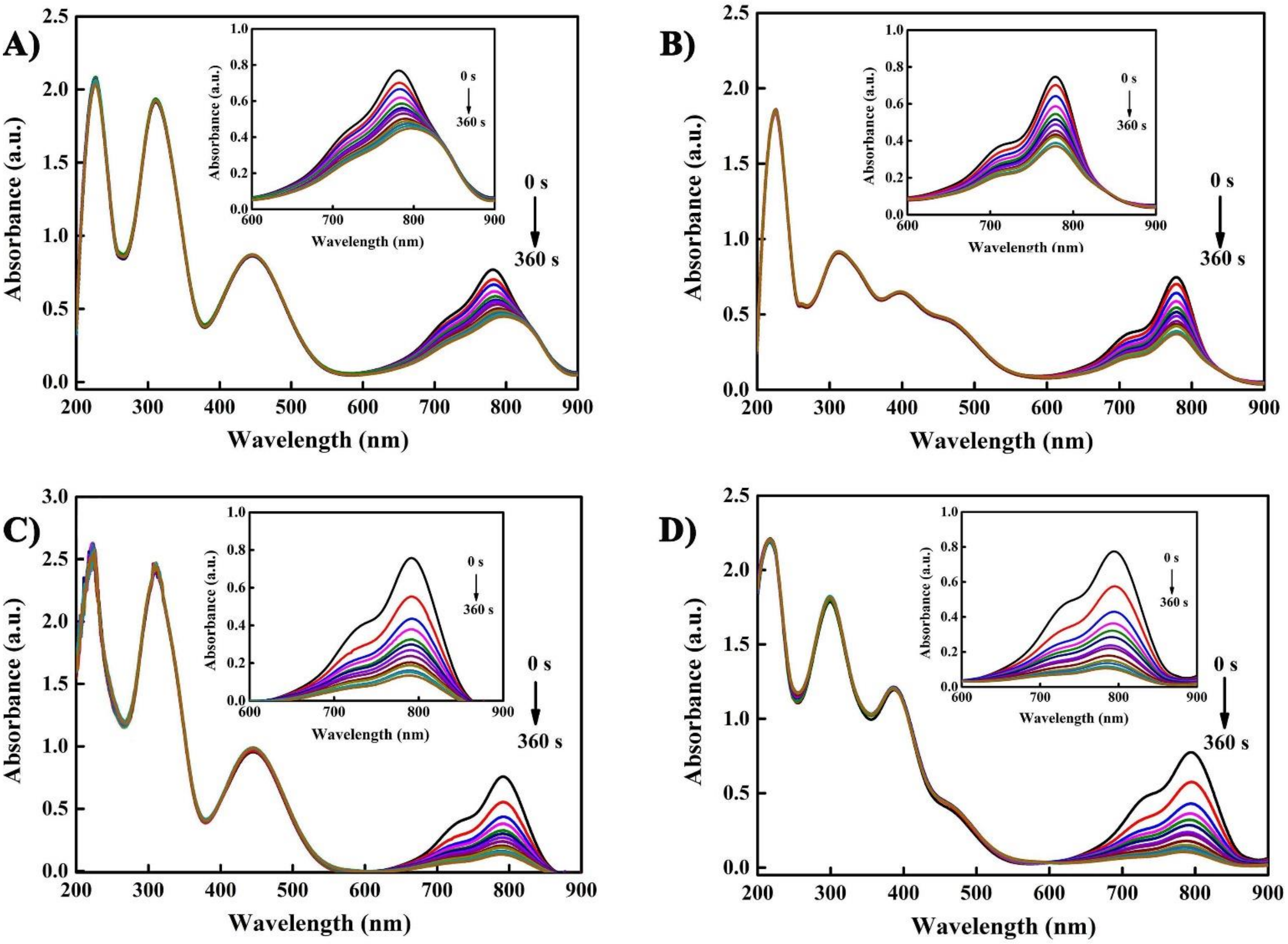

Figure S17. UV–vis absorption spectra of ICG (5 μg mL^{-1}) in the presence of A) **PS1**, B) **PS2**, C) **PS1** NPs and D) **PS2** NPs (30 μg mL^{-1}) for different times under white irradiation (20 mW cm^{-2}).

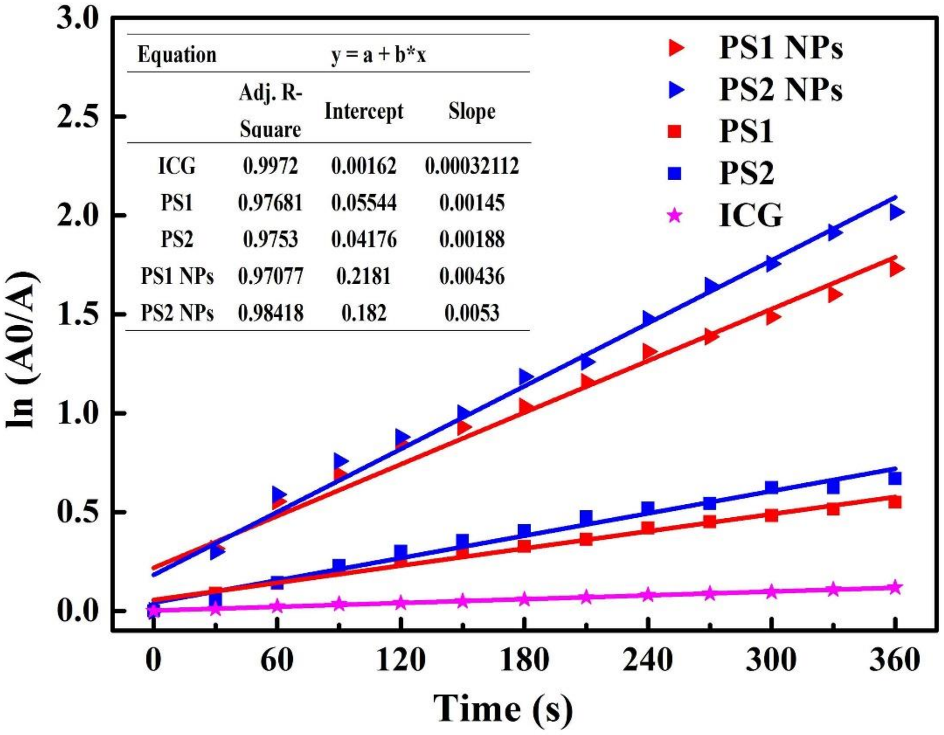

Figure S18. Time-dependent $^1O_2$ generation kinetics. A_0 = absorption of ICG without irradiation. A = real-time absorption of ICG with different irradiation time.

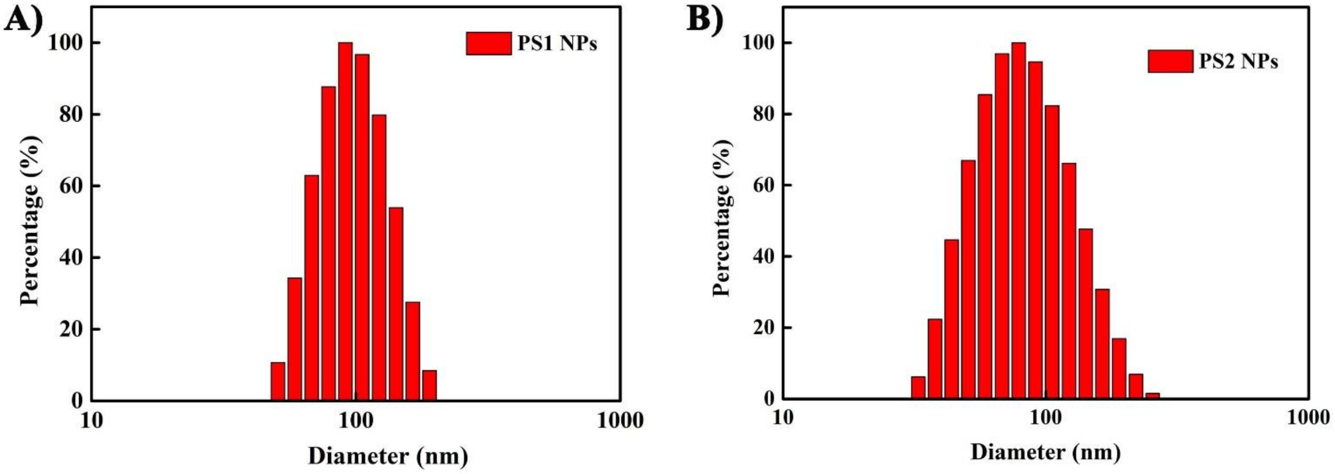

Figure S19. Dynamic laser scattering results of A) **PS1** NPs and B) **PS2** NPs in water.

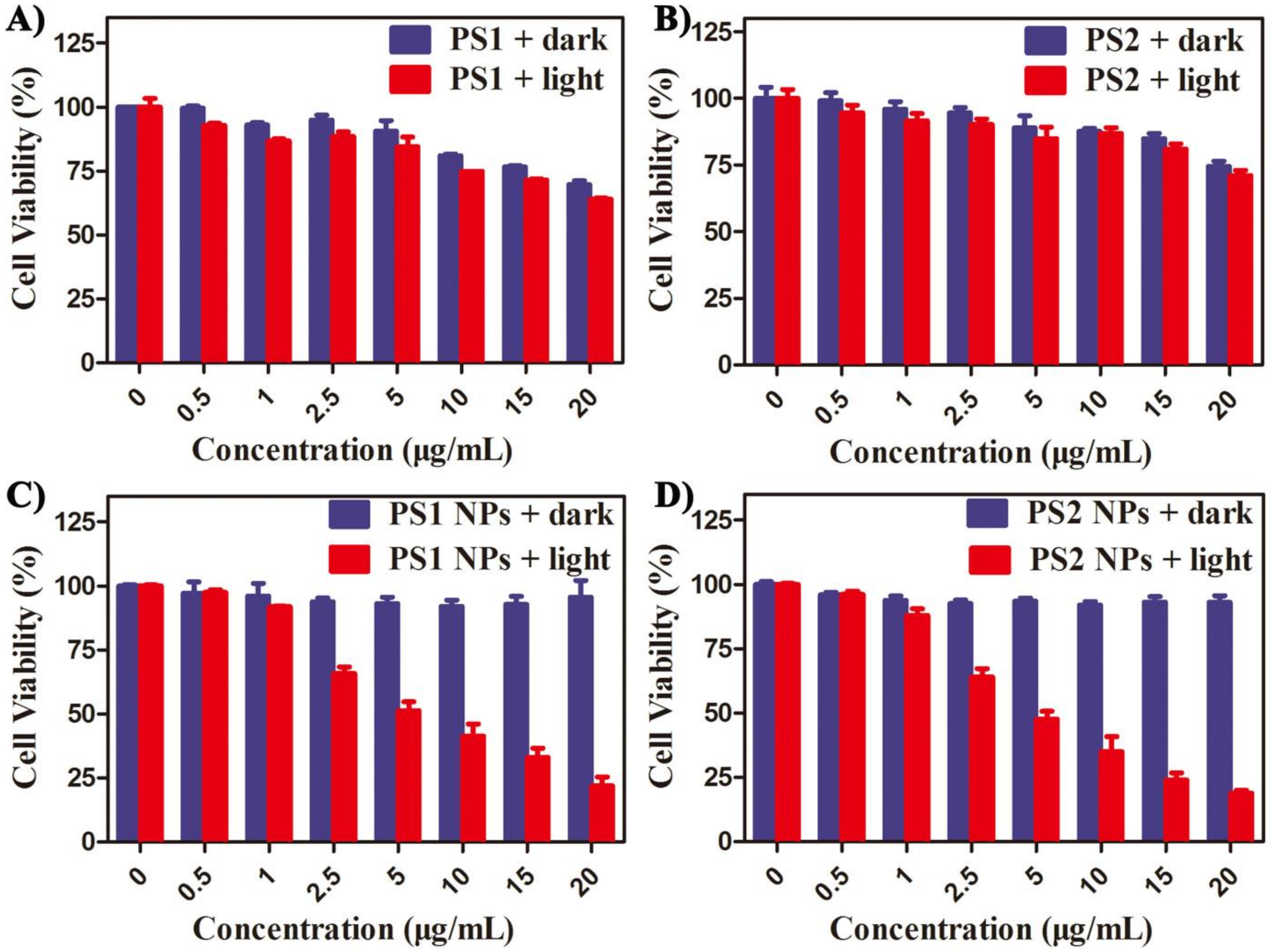

Figure S20. Viability of HeLa cells treated with A) **PS1**, B) **PS2**, C) **PS1** NPs, and D) **PS2** NPs with or without white light (20 mW cm^{-2}, 60 min).

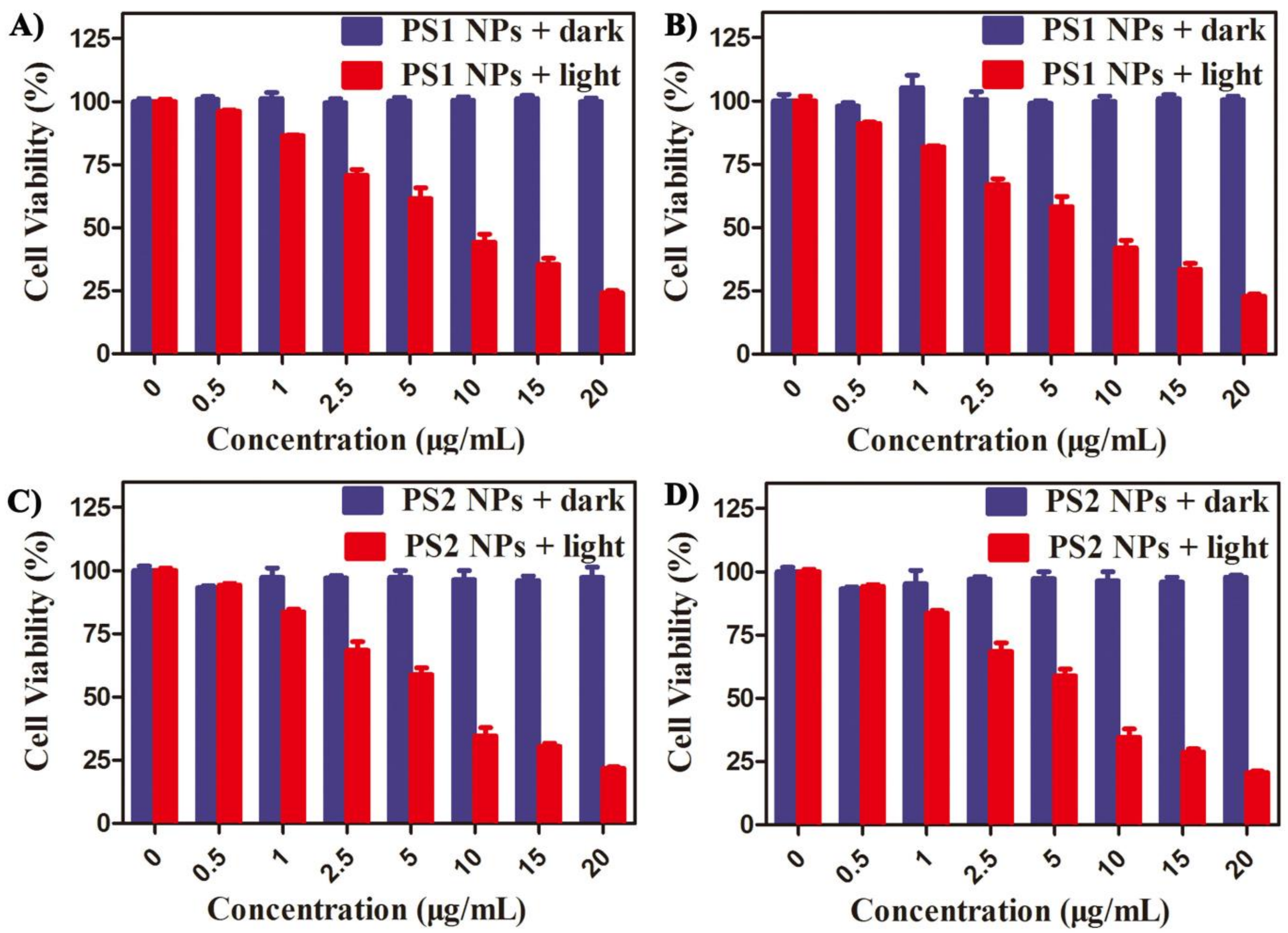

Figure S21. Viability of A549 cells treated with A) **PS1** NPs and C) **PS2** NPs with or without white light (20 mW cm^{-2}, 60 min). Viability of MDA-MB-231 cells treated with B) **PS1** NPs and D) **PS2** NPs with or without white light (20 mW cm^{-2}, 60 min).

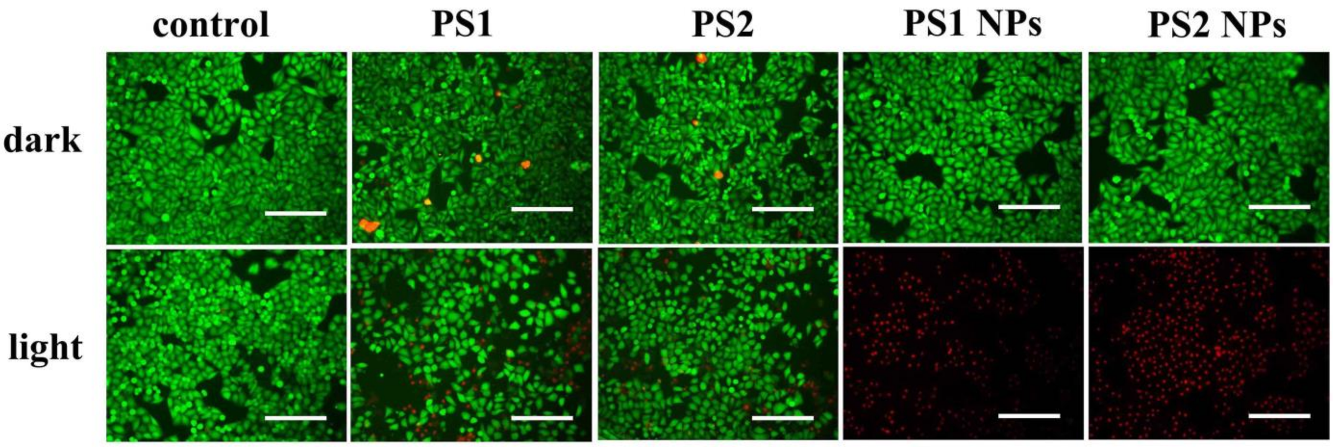

Figure S22. Live/dead cell of different PSs (20 μg mL^{-1}) staining was tested by using calcein-AM (green fluorescence for live cells) and propidium iodide (PI, red fluorescence for dead cells). The scale bars are 40 μm.

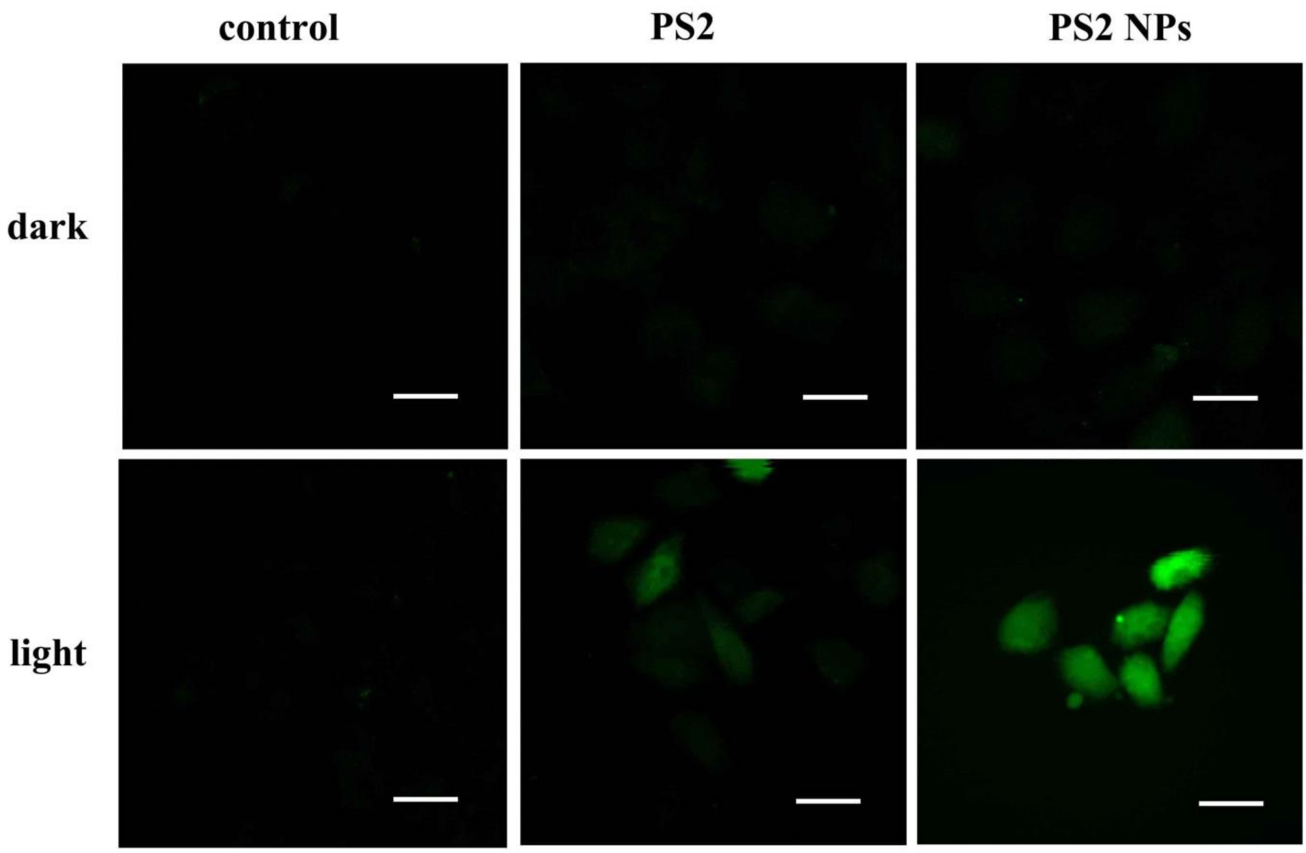

Figure S23. Generation of intracellular ROS mediated by **PS2** and **PS2** NPs (10 μg mL^{-1}) upon white light irradiation (20 mW cm^{-2}, 20 min) as indicated by the fluorescence of DCF. Scale bar = 20 μm for all images.

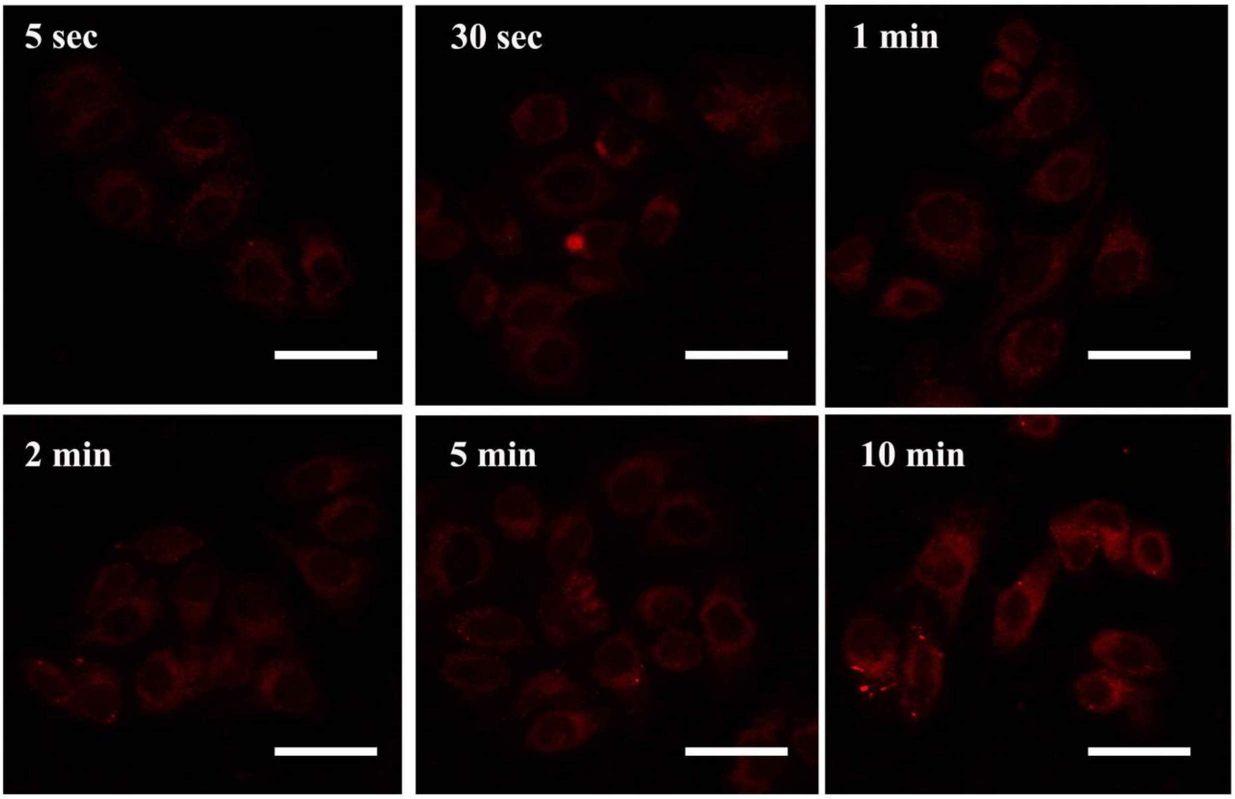

Figure S24. CLSM images of HeLa cells after incubation with **PS1** NPs (0.5 µg mL^{-1}) for different times. Scale bar = 20 µm for all images.

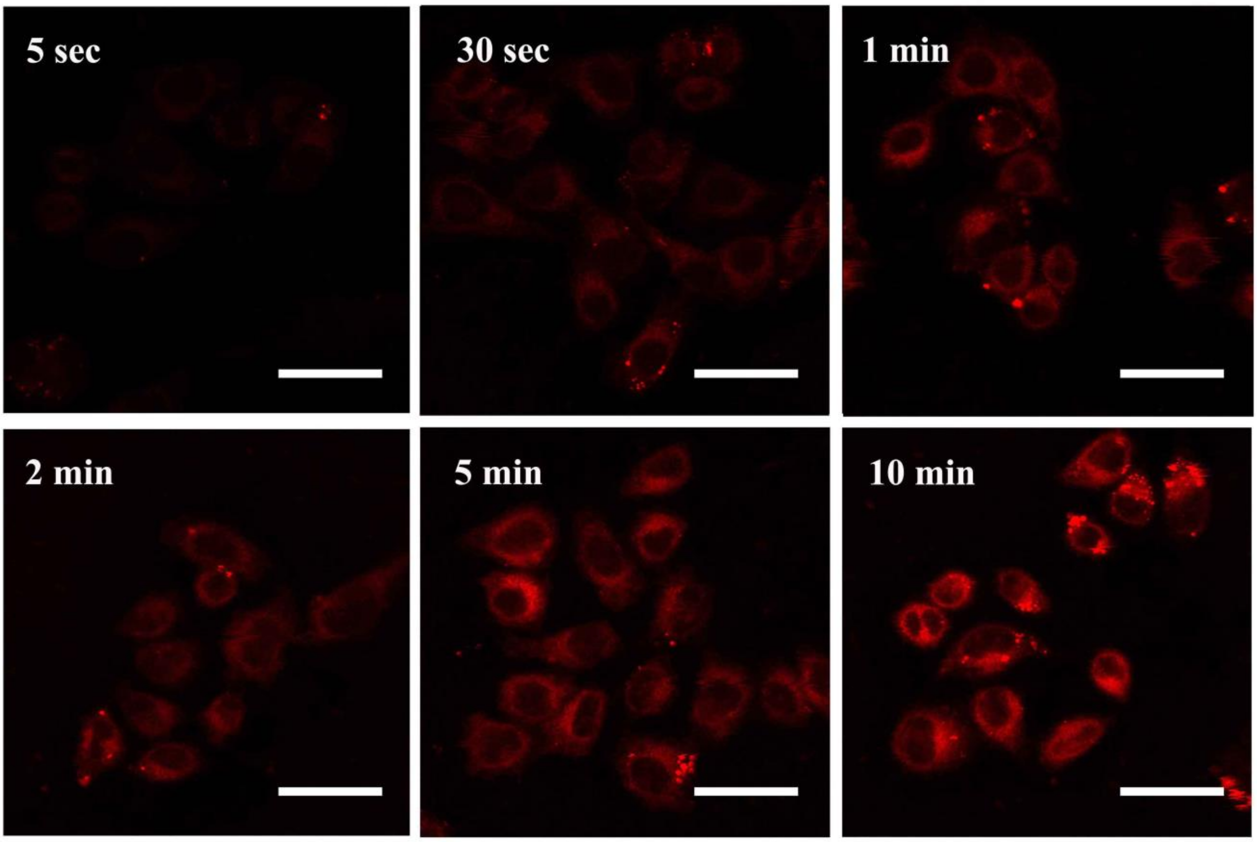

Figure S25. CLSM images of HeLa cells after incubation with **PS1** NPs (1 μg mL^{-1}) for different times. Scale bar = 20 μm for all images.

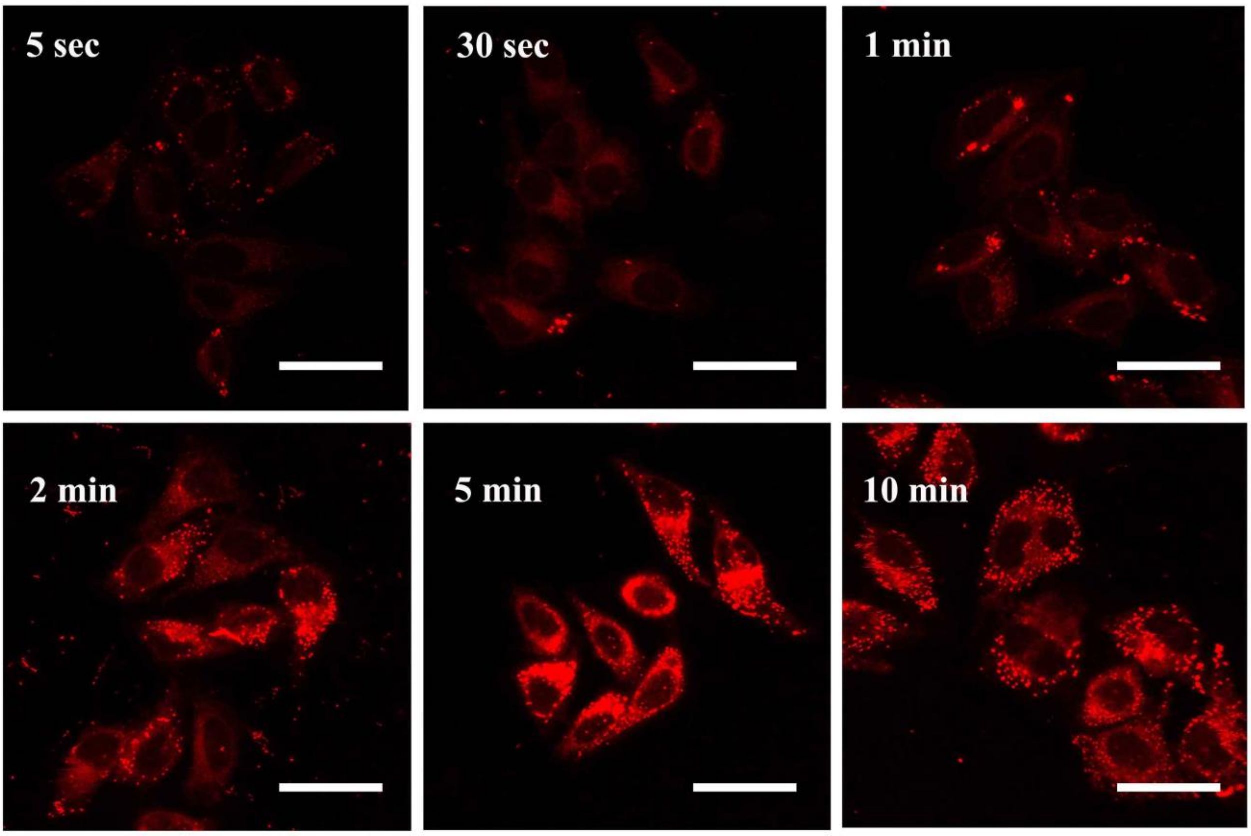

Figure S26. CLSM images of HeLa cells after incubation with **PS1** NPs (2 μg mL^{-1}) for different times. Scale bar = 20 μm for all images.

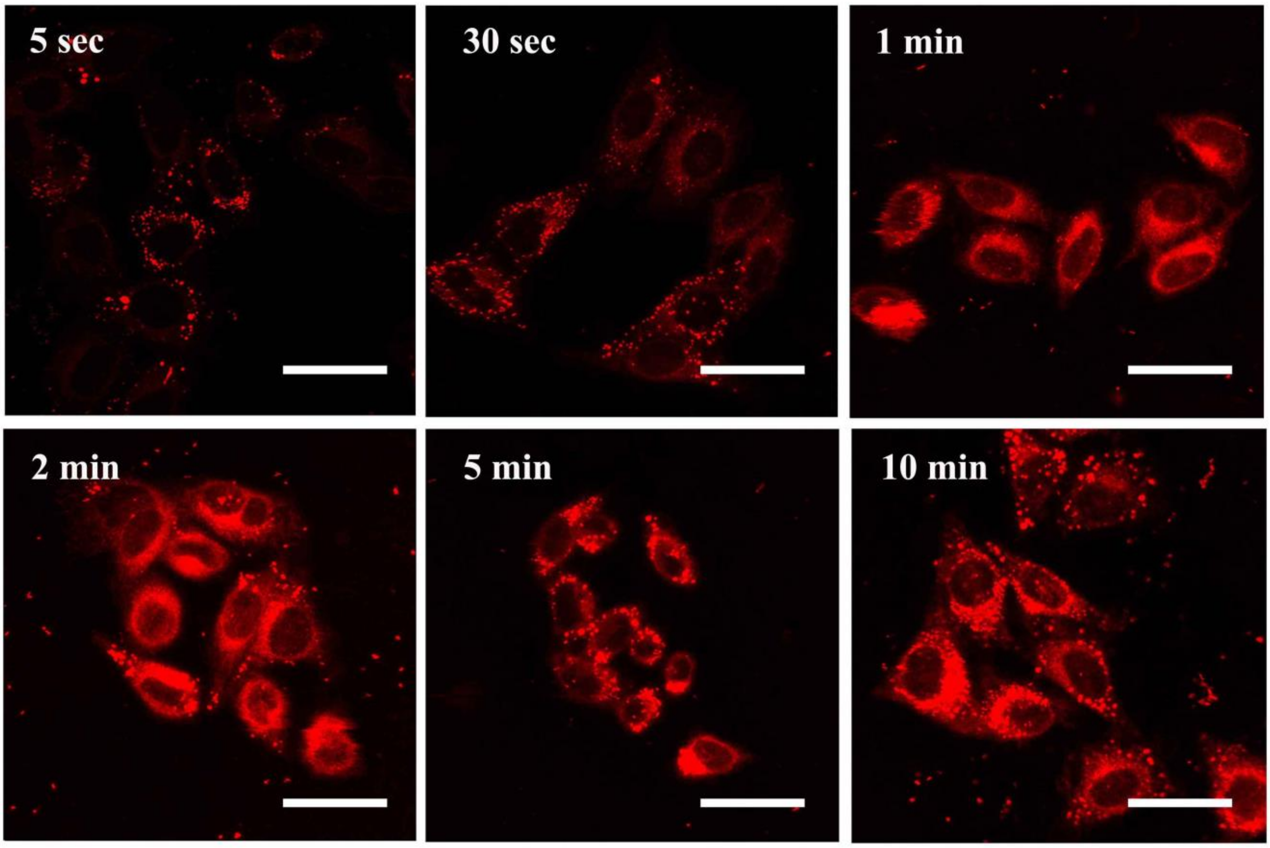

Figure S27. CLSM images of HeLa cells after incubation with **PS1** NPs (3 μg mL^{-1}) for different times. Scale bar = 20 μm for all images.

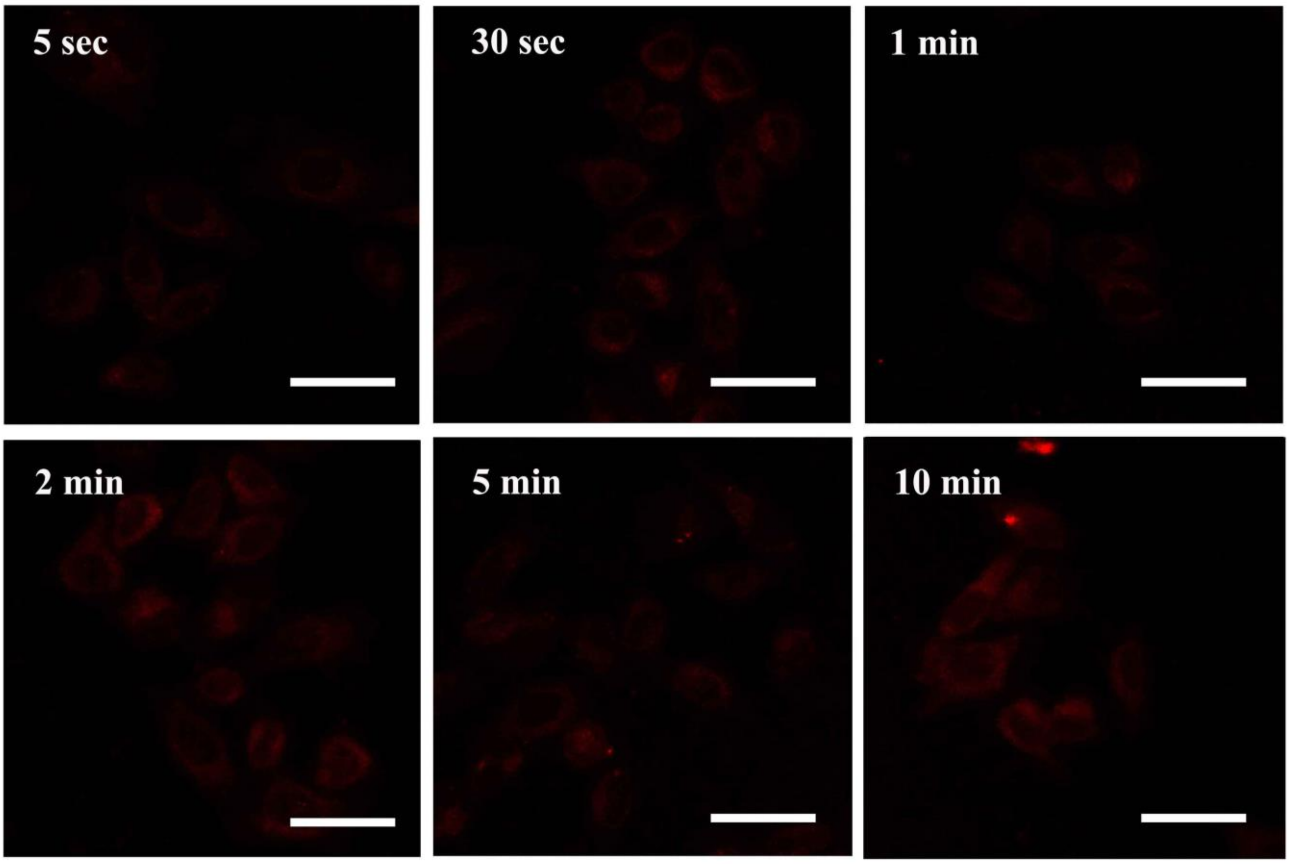

Figure S28. CLSM images of HeLa cells after incubation with **PS2** NPs (3 μg mL^{-1}) for different times. Scale bar = 20 μm for all images.

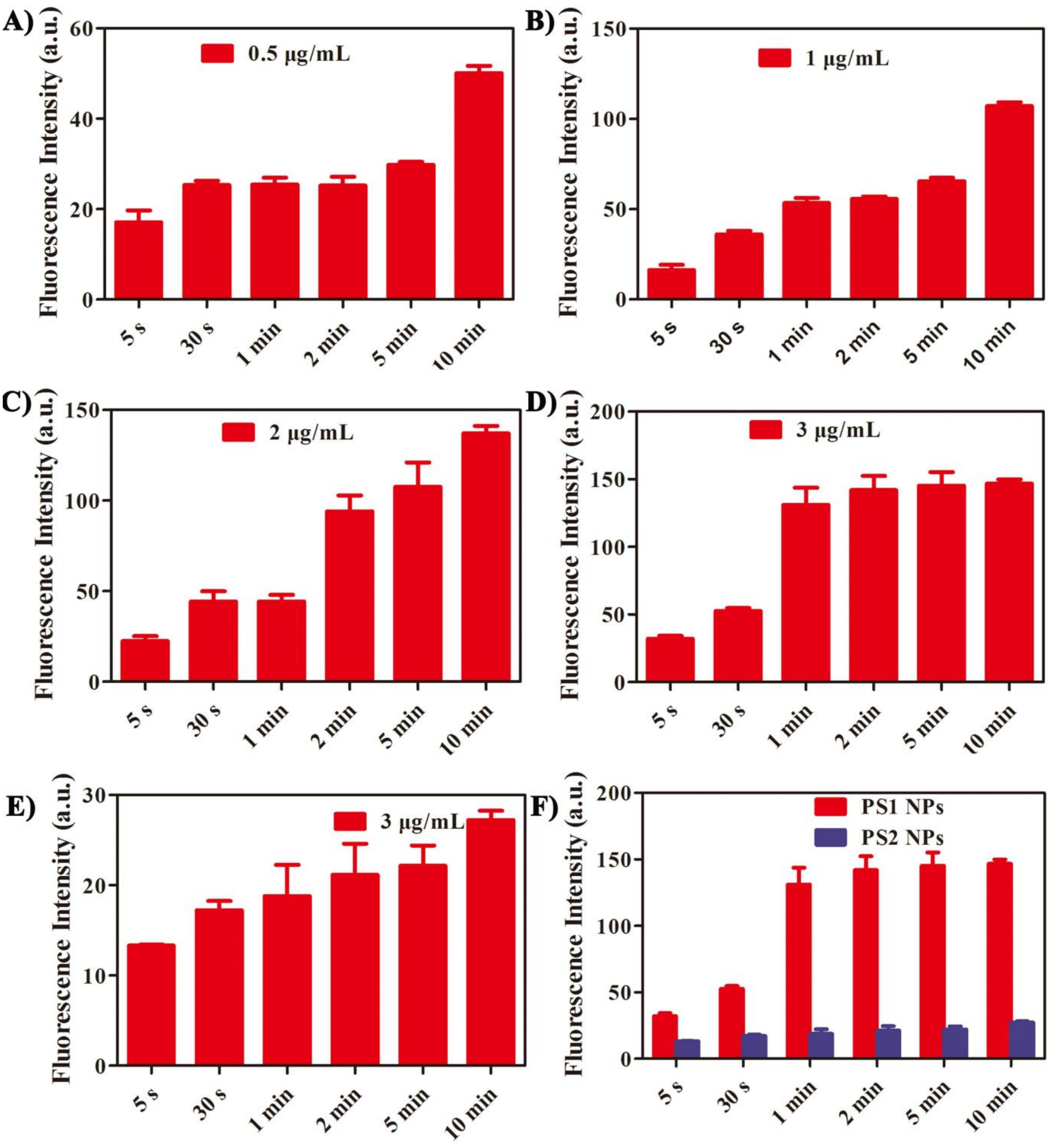

Figure S29. Time-dependent fluorescence intensity changes of HeLa cells after incubation with A) **PS1** NPs (0.5 µg mL^{-1}), B) **PS1** NPs (1 µg mL^{-1}), C) **PS1** NPs (2 µg mL^{-1}), D) **PS1** NPs (3 µg mL^{-1}), E) **PS2** NPs (3 µg mL^{-1}), F) **PS1** NPs (3 µg mL^{-1}) and **PS2** NPs (3 µg mL^{-1}).

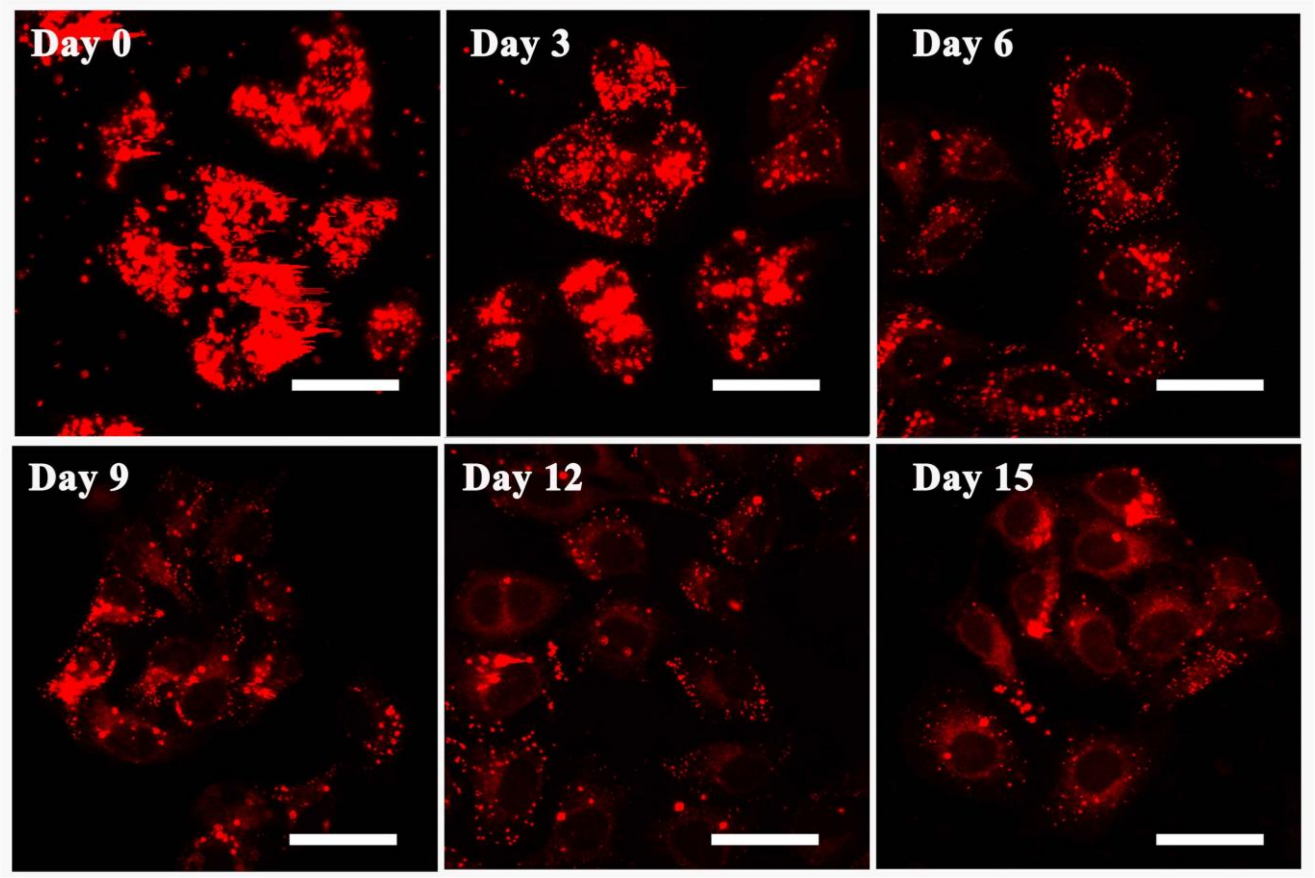

Figure S30. Long-term cell tracing images of the **PS1** NPs (20 μg mL^{-1}) at 37 °C for 6 h and then subcultured for different days. Scale bar = 20 μm for all images.

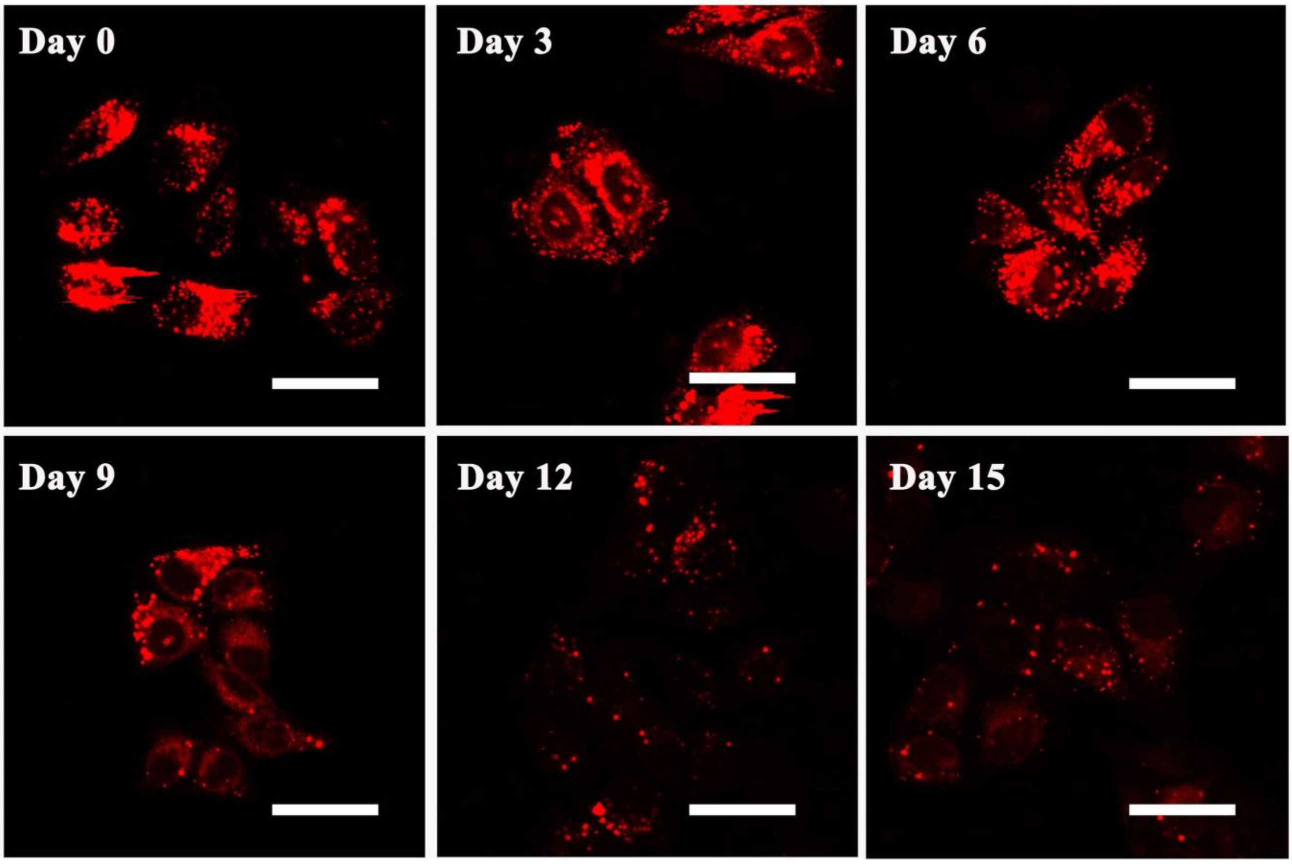

Figure S31. Long-term cell tracing images of the **PS2** NPs (20 μg mL^{-1}) at 37 °C for 6 h and then subcultured for different days. Scale bar = 20 μm for all images.

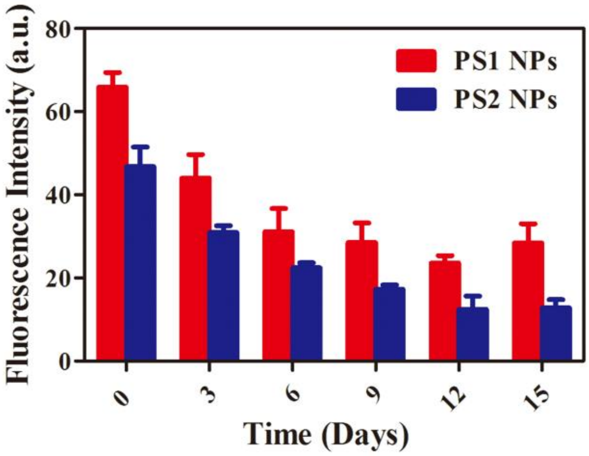

Figure S32. Time-dependent fluorescence intensity changes of long-term cell tracing of the **PS1** NPs and **PS2** NPs for different days.

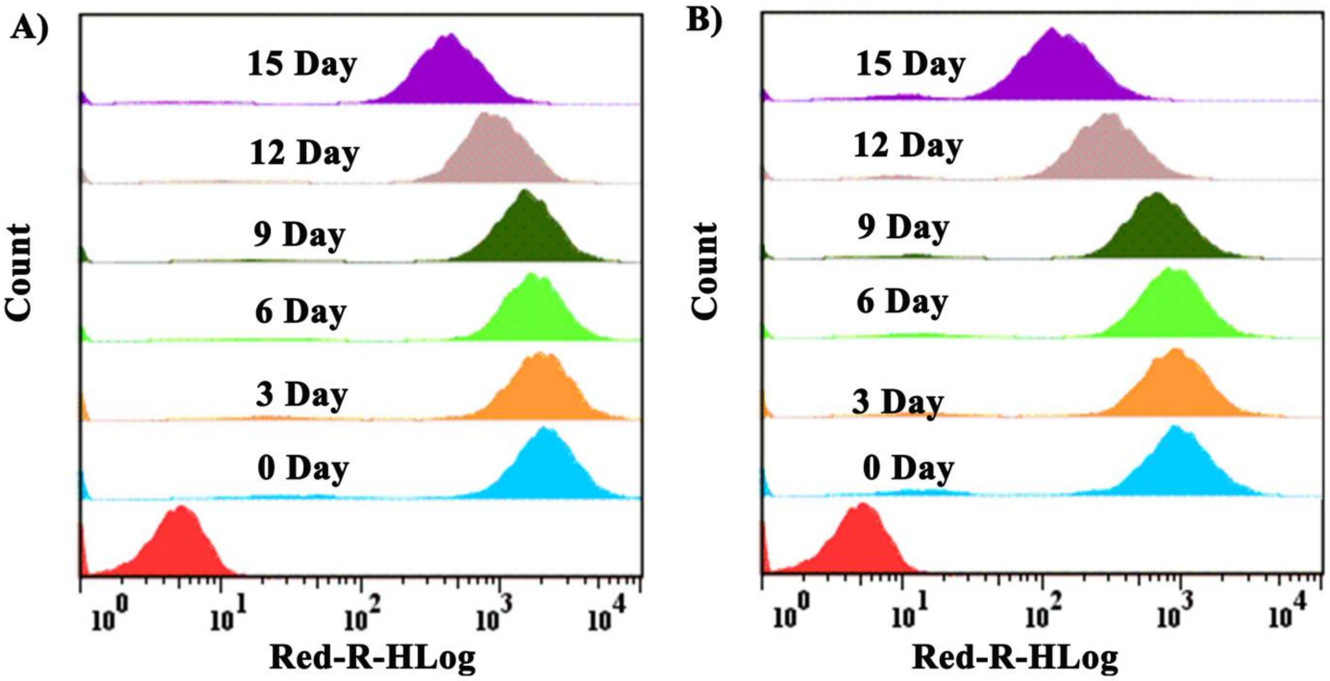

Figure S33. Flow cytometry analysis of long-term cell tracing of the **PS1** NPs and **PS2** NPs for different days.

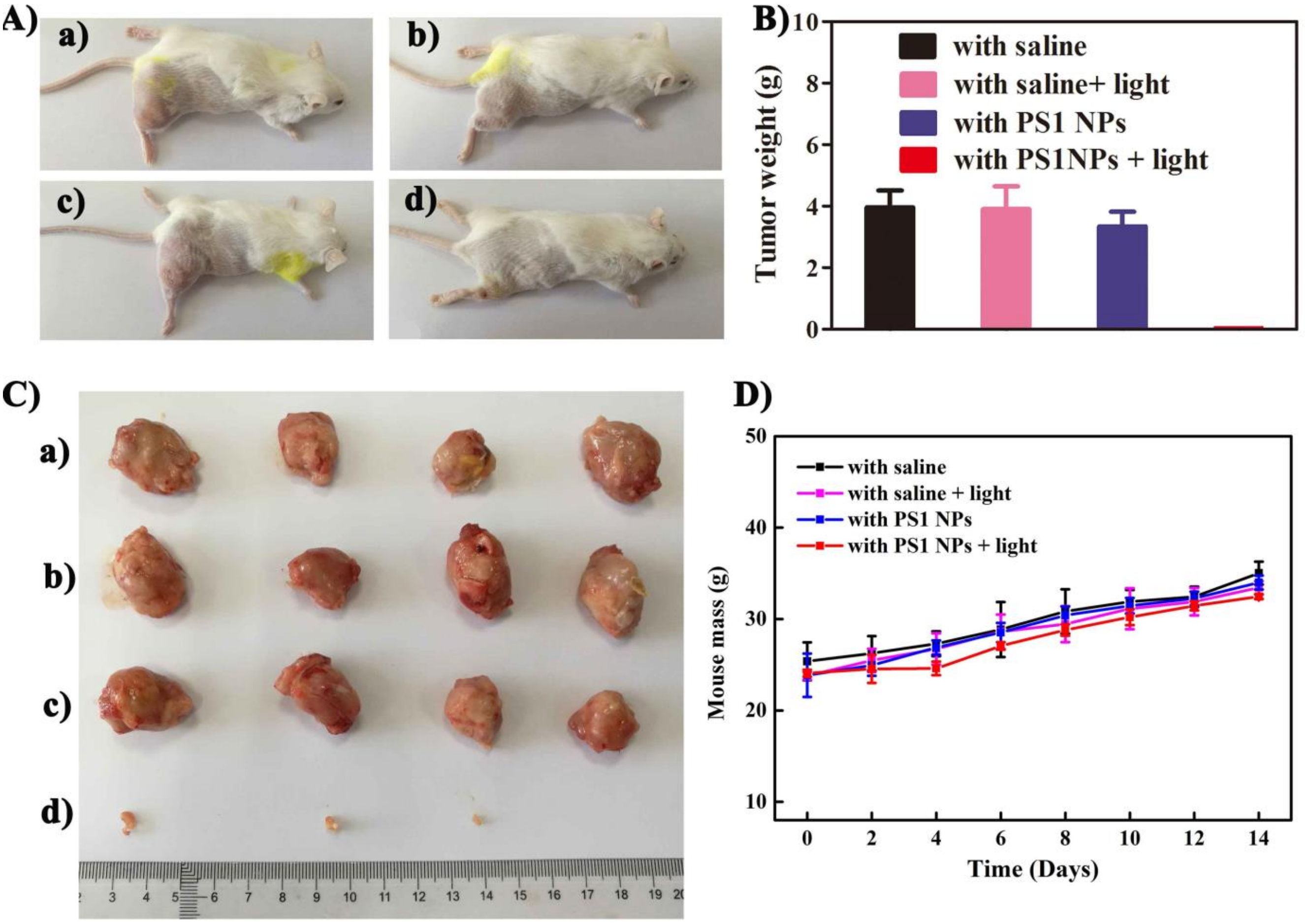

Figure S34. A) Representative images of mice. B) Tumor weights of four groups. C) Harvested tumors from various groups treated (a) with saline, (b) with saline and light, (c) with **PS1** NPs, (d) with **PS1** NPs and light (100 μg mL^{-1}, 100 μL), white light irradiation (200 mW cm^{-2}, 20 min). D) Body weights of mice for different groups of mice.

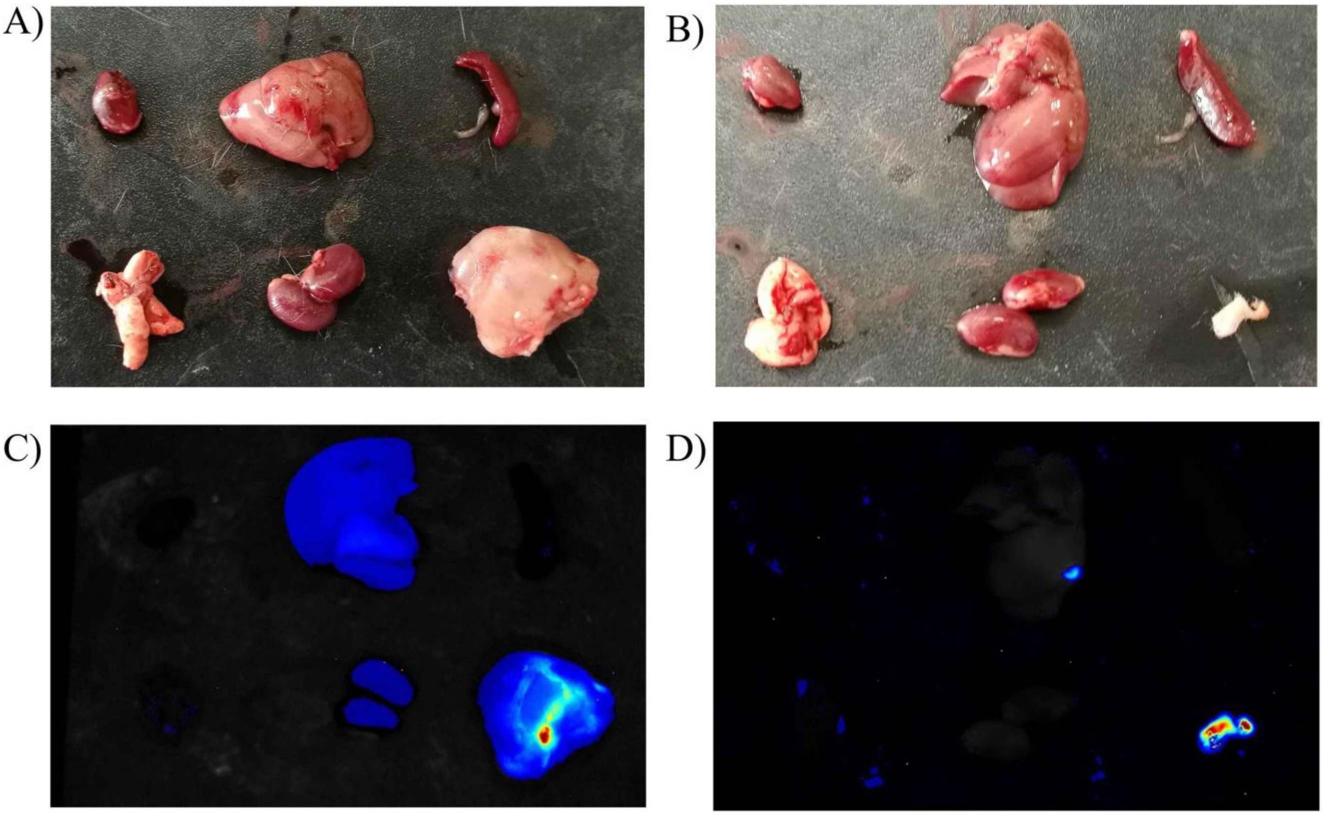

Figure S35. Fluorescence of major tissues of mice after intravenous injection of A,C) with **PS1** NPs and B,D) with **PS1** NPs + light for 14 days.

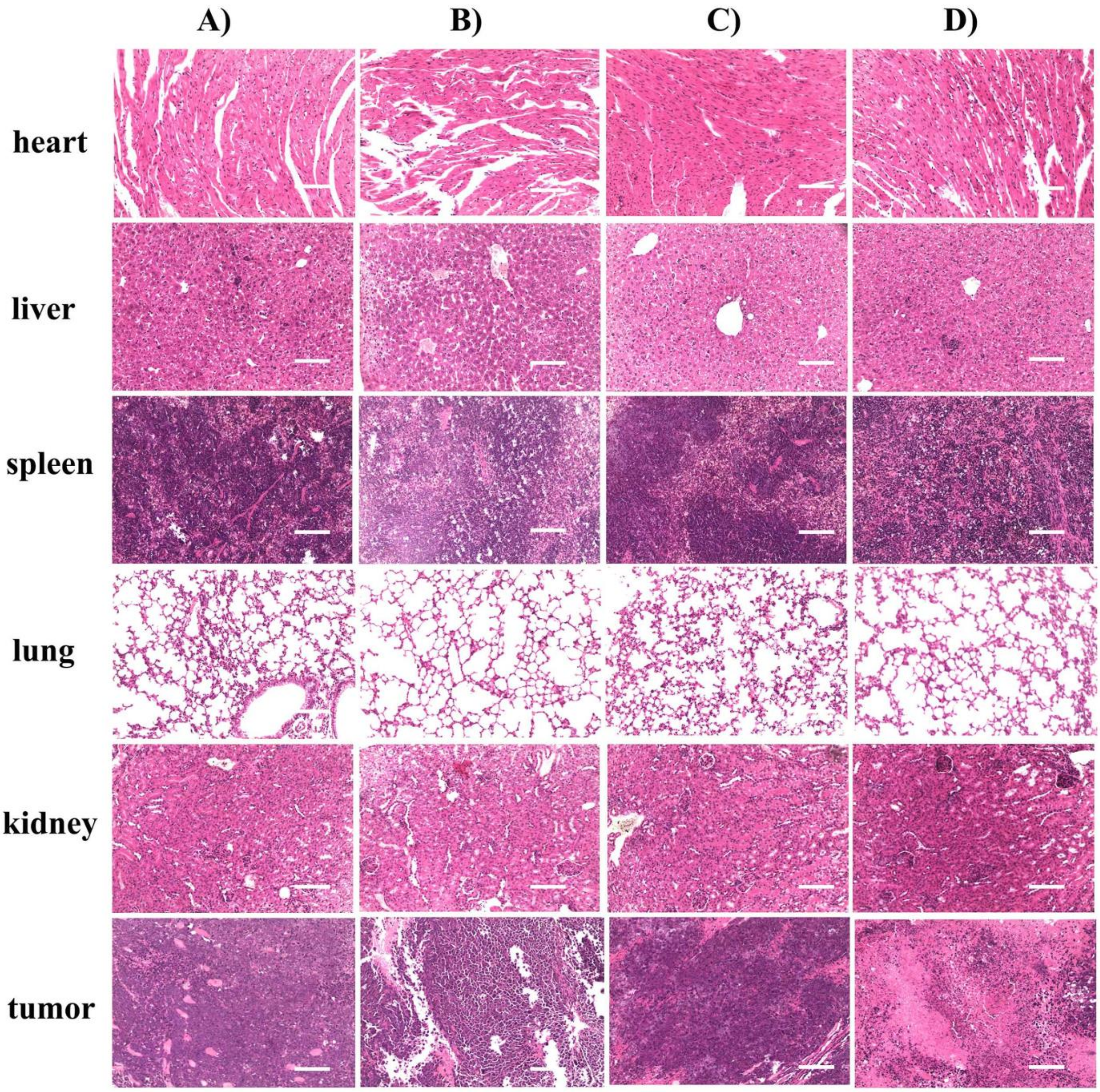

Figure S36. H&E staining of various organs from mice at the end of experiments after treatment a) with saline, b) with saline and light, c) with **PS1** NPs, d) with **PS1** NPs and light.

Table S1. Photophysical data of **PS1**, **PS2** and the corresponding NPs.

	λ_{abs} (nm)	λ_{em} (nm)	Φ_F(%)	τ_F (ns)	k_r ^{d}($\times10^4$ s^{-1})	k_{nr} ^{d}($\times10^4$ s^{-1})
**PS1**[a]	331; 446	660	16	8.57	1.866	9.801
**PS2**[a]	312; 394; 465	645	13	21.23	0.612	4.098
**PS1** NPs[b]	331; 446	659	40	15.58	2.567	3.851
**PS2** NPs[b]	312; 394; 465	644	32	29.34	1.091	2.317

[a] Measured in DMSO/water (v/v = 1:9) at 298 K (1.0×10^{-5} M, λ_{ex} = 469 nm). [b] Measured in water at 298 K (1.0×10^{-5} M, λ_{ex} = 488 nm).

Table S2. The average diameter and polydispersity index (PDI) results of **PS1**/ **PS2** NPs measured by DLS.

Sample	**PS1** NPs	**PS2** NPs
Average diameter (nm)	82	79
PDI	0.054	0.103

[a] Measured in DMSO/water (v/v = 1:9) at 298 K (1.0×10^{-5} M, λ_{ex} = 469 nm). [b] Measured in water at 298 K (1.0×10^{-5} M, λ_{ex} = 488 nm).

Table S3. The drug loading content (DLC) and drug loading efficiency (DLE) results of **PS1/ PS2** NPs.

Sample	**PS1** NPs	**PS2** NPs
DLC (mg/mL)	0.1355	0.1438
DLE (%)	90.79	96.35

4. Reference for the SI.

1. M. J. Frisch, et al., Gaussian09 (RevisionD.01), Gaussian, Inc., Wallingford, Connecticut, 2009.
